# Design, in silico studies and biological evaluation of novel chalcones tethered triazolo[3,4-*a*]isoquinoline as EGFR inhibitors targeting resistance in non-small cell lung cancer

**DOI:** 10.1038/s41598-024-76459-x

**Published:** 2024-11-04

**Authors:** Nesma Abdelaal, Mohamed A. Ragheb, Hamdi M. Hassaneen, Emad M. Elzayat, Ismail A. Abdelhamid

**Affiliations:** 1 Biotechnology Department, Faculty of Science, Cairo University, Cairo, Egypt; 2 Department of Chemistry (Biochemistry Division), Faculty of Science, Cairo University, Giza, 12613 Egypt; 3 Chemistry Department, Faculty of Science, Cairo University, Cairo, Egypt; 4 Zoology Department, Faculty of Science, Cairo University, Cairo, Egypt

**Keywords:** Chalcones, Triazolo[3,4-*a*]isoquinoline, 1,3-Diphenyl-1*H*-pyrazole, NSCLC, Apoptosis, EGFR inhibitors, Anticancer activity, Biochemistry, Cancer

## Abstract

A novel series of six [1,2,4]triazolo[3,4-*a*]isoquinolin-3-yl)-3-(1,3-diphenyl-1*H*-pyrazol-4-yl)prop-2-en-1-ones (**3a**–**3f**) was designed and synthesized. They were characterized based on spectral and elemental analyses. In silico studies were also committed to provide insights and a better understanding of their structural features. The six compounds were screened for their antiproliferative activity using the MTT assay against five human cancer cell lines, namely, A549, HCT116, PC3, HT29, and MCF-7 in parallel with the non-cancerous human lung cell line WI-38. The results showed that **3e** and **3f** have potential cytotoxic activities, especially on A549 cells with IC_50_ = 2.3 µM and 1.15 µM, respectively. Meanwhile, they recorded a minimal cytotoxic effect on WI-38 cells. Concerning the molecular mechanism of action, the present study showed the inhibitory effect of the six compounds against total EGFR. The most potent EGFR inhibitors were **3e** and **3f** with IC_50_ = 0.031 µM and 0.023 µM, respectively. The selectivity index of **3f** for EGFR^T790M^ was 1.81 times more selective than that of lapatinib. In addition, **3e** and **3f** initiated cell cycle arrest at the G2/M and pre-G1 phases along with the downregulation of anti-apoptotic protein Bcl2 and the upregulation of pro-apoptotic proteins: p53, Bax, and caspases 3, 8, and 9. Further studies are recommended to evaluate animal models’ promising anticancer activity and molecular mechanism of triazolo[3,4-*a*]isoquinoline derivatives **3e** and **3f**.

**Supplementary Information:**

The online version contains supplementary material available at 10.1038/s41598-024-76459-x.

## Introduction

**Fig. 1:**
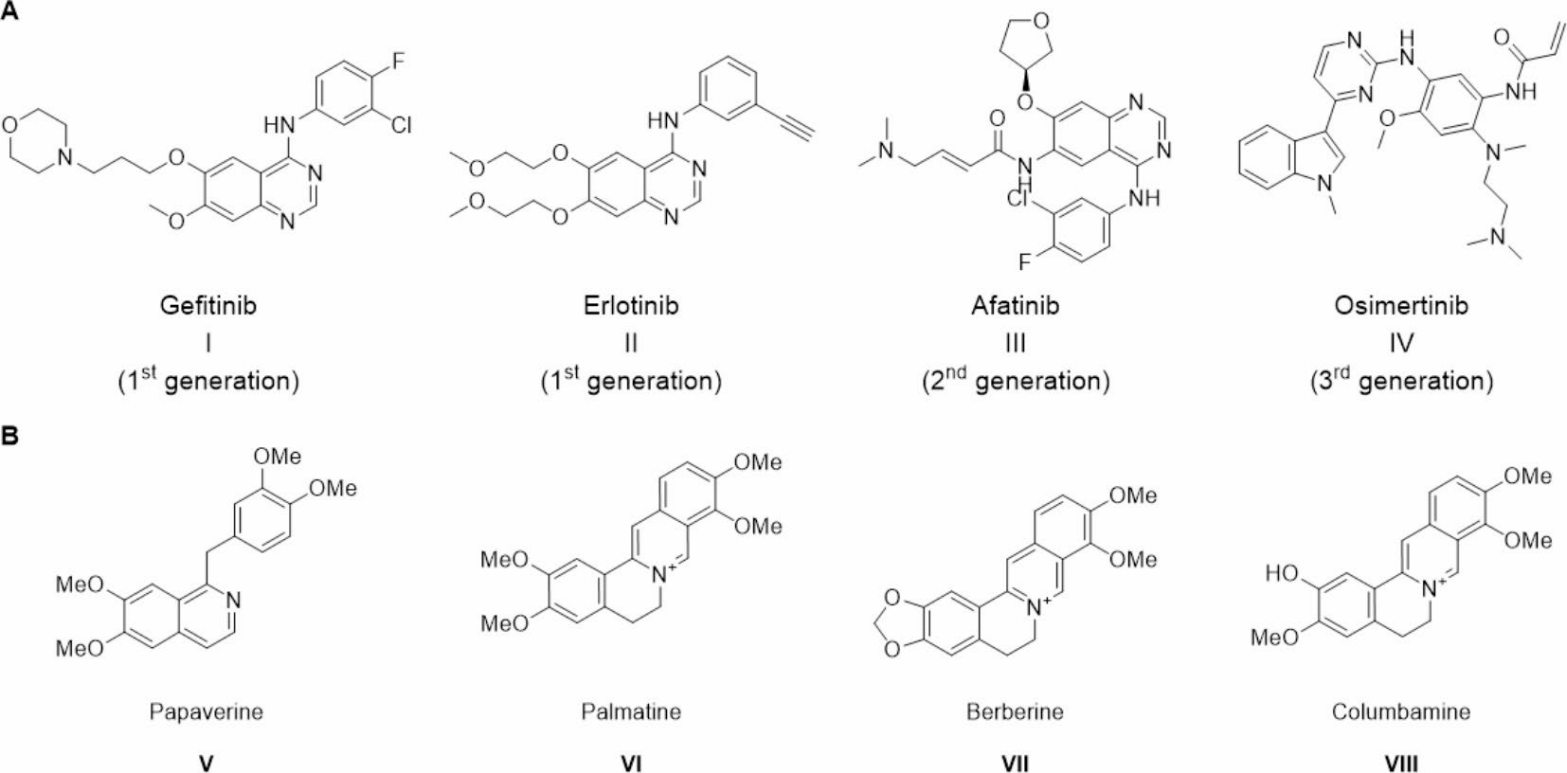
Examples of some reported EGFR inhibitors (**A**) and bioactive alkaloids containing isoquinoline moiety (**B**).

Protein kinases play critical roles in signaling pathways that mediate multiple cellular functions. Since they catalyze the transfer of the gamma-phosphate group from an ATP to targeted proteins^1^. Protein kinase inhibitors have been an attractive target for chemotherapeutics over the past decades since they are directly responsible for tumor cells’ growth, differentiation, and survival. At present, carcinoma is among the most fatal and aggressive illnesses, contributing significantly to death rates. Over 19.3 million new cases of malignancies were recently found and documented; according to the data given, over 10 million individuals died from cancer in 2020^2,3^. Human carcinomas frequently express high levels of receptors belonging to the epidermal growth factor (EGF) receptor family^4^. EGFR (also known as ErbB-1/HER1) is a 170 kDa transmembrane glycoprotein and is considered a member of the ErbB family of cell membrane receptors. The EGFR consists of three domains, the extracellular domain: which recognizes and binds to specific ligands, the hydrophobic transmembrane domain which is elaborated in interactions between receptors within the cell membrane, and the intracellular domain which contains the tyrosine kinase (TK) enzymatic activity^5^. When a ligand binds to the extracellular domain, EGFR is homodimerized or heterodimerized. Dimerization induces activation of the TK domain leading to autophosphorylation of a key tyrosine residue in the cytoplasmic tail. These tyrosine residues serve as binding sites for cellular proteins that activate various downstream signaling pathways^6^. EGFR mediates multiple signaling cascades (Ras/Raf mitogen-activated protein kinase, phosphoinositide-3-Kinase (PI3K)/Akt, and Jak2/STAT3) which commutatively regulate cell proliferation, apoptosis, angiogenesis, invasion, and metastasis. EGFR is commonly expressed in various epithelial tumors such as breast, colon, ovarian, prostate, and non-small cell lung cancer (NSCLC)^7,8^. EGF system ligands and receptors are over-expressed by 40–80% in NSCLC^9^. The overexpression of EGFR and closely related ErbB2 have been associated with more aggressive cancer symptoms. It has been proved that EGFR inhibitors have a positive effect on the induction of apoptosis, inhibition of invasion, and metastasis. It enhances the antitumor activity of cytotoxic drugs and radiotherapy^10^. Monoclonal antibodies and small-molecule inhibitors have been successful pharmacological approaches targeting EGFR TK enzymatic activity. Small molecules inhibit EGFR autophosphorylation and downstream signaling cascades such as Gefitinib **I** and Erlotinib **II** (Fig. 1A)^11^. Erlotinib is a quinazoline derivative inhibitor of the EGFR TK activity, that is used in the treatment of non-small cell lung cancer (NSCLC), pancreatic cancer, and several other types of cancer. Erlotinib binds to the EGFR extracellular domain reversibly at the adenosine triphosphate (ATP) binding site of the receptor. Gefitinib is an EGFR inhibitor, like erlotinib, which interrupts signaling through the epidermal growth factor receptor (EGFR) in target cells^12^.

Activating mutations in the EGFR gene left a tremendous impact on treatment procedures in NSCLC. To overcome such mutations, the recent discovery of new EGFR inhibitors played an important role^13^. EGFR enzymes have certainly been undergoing missense mutations which lead to resistance. Changes in the functions of the protein have forced researchers to discover new drugs to avoid as well as inhibit resistance^14^. EGFR mutations are classified by nucleotide changes. The first mutation was found in the form of an Exon19 deletion^15^. Drugs such as erlotinib and gefitinib, which are widely used to treat lung cancer, have induced such resistance. Mutational emergences have provided new targets and compounds, named in terms of generations. Drugs that inhibit target enzymes were considered the first-generation^16^.

Continuing a similar quinazoline scaffold, second-generation drugs were designed by changing the residing R-groups. Neratinib and afatinib **III** (Fig. 1A) exhibit inhibitory activity towards the Exon19 deletion^17^. Until the wild-type, the EGFR enzyme showed a new missense mutation in the form of the replacement of a specific AUG gene with the UAU gene^18^. This resulted from a change in the amino acid of threonine to methionine at position 790. The EGFR T790M mutation also altered the binding possessions functions which were pragmatic in the form of toxicity^19^. To overcome the mutations and observed toxicity, new drugs have emerged with a scaffolding change from the quinazoline ring to the pyrimidine ring^20^. The most recent development in the form of third-generation molecules was rociletinib and Osimertinib **IV** (Fig. 1A) which showed improved action against mutations such as exon deletion and T790M mutation^21^.

Heterocycles containing bridgehead nitrogen atoms, such as fused isoquinolines, are important because they represent the key structures of several alkaloids, including papaverine^22^, columbamine^23^, palmatinem^23^, berberine^24^. Furthermore, they have a wide range of biological properties, such as antidepressant^25,26^, anticancer^27–29^, cardiovascular^26^, anti-inflammatory^26,30,31^, antimalarial^32^, and anti-HIV^33^ activity. Isoquinolines are of great significance since they represent the basic structural elements of numerous alkaloids, such as papaverine **V**^22^, palmatinem **VI**^23^, berberine **VII**^24^, and columbamine **VIII**^23^ (Fig. 1B).

**Fig. 2:**
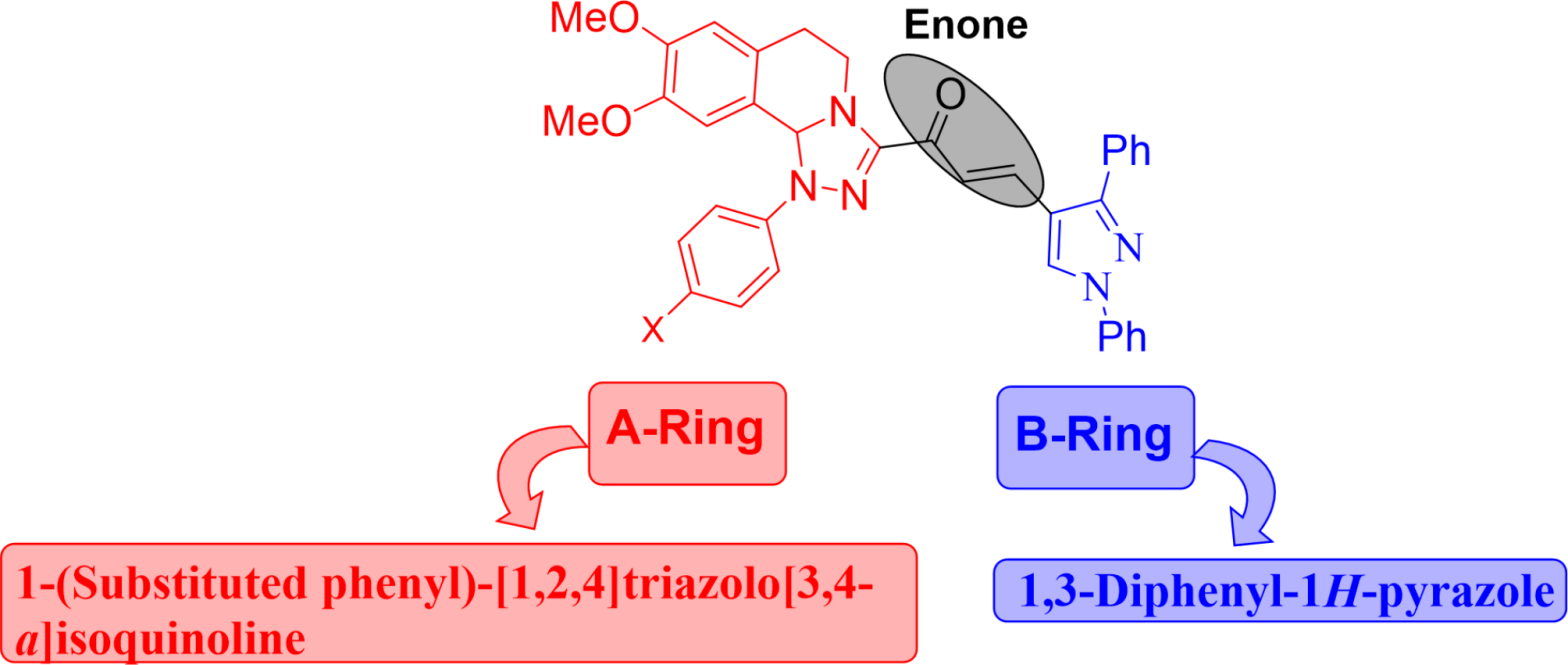
The design strategy of the newly synthesized [1,2,4]triazolo[3,4-*a*]isoquinolin-3-yl)-3-(1,3-diphenyl-1*H*-pyrazol-4-yl)prop-2-en-1-ones compounds.

By attaching different active groups to the isoquinoline moiety through various synthetic protocols, researchers have been able to demonstrate a wide range of biological activities^34^. Molecular hybridization (MH) is recognized as an effective strategy for the synthesis of molecules that have several structural units and increased bioactivities. The idea of hybrid pharmaceuticals has provided a new drug design technique that combines two or more drugs with intrinsic action in one agent^35,36^. Depending on the aforementioned background about EGFR inhibitors and their relation to cancer therapy^37–40^, and in continuation to our research interest in the preparation of biologically active chalcones^28,41–52^, and heterocycles^53–63^ in the present study, we combined all these structural units in one hybrid molecule through the synthesis of novel chalcones incorporating [l,2,4]triazolo[3,4-*a*]isoquinoline and 1,3-diphenyl-1*H*-pyrazole scaffolds with different electron-donating and electron-withdrawing substituents, as illustrated in Fig. 2. The designed hybrid chalcones incorporating triazolo[3,4-a]isoquinoline and 1,3-diphenyl-1 H-pyrazole moieties share many similarities with those traditional EGFR inhibitors (Fig. 1) because they are based on the essential pharmacophoric features of EGFR inhibitors in targeting the ATP-binding site of the EGFR tyrosine kinase domain. These similarities include the possession of heterocyclic cores, planar or aromatic systems, hydrophobic interactions, and hydrogen bond-forming capabilities, all of which may confer unique interactions, improved selectivity, enhanced binding affinity in EGFR inhibition, or overcome the resistance raised by previous drugs. Therefore, the designed chalcone derivatives were subjected to molecular docking to understand binding pockets and predict their potency. Finally, the designed compounds were synthesized and subjected to various biological evaluations, including cytotoxic activities, cell cycle arrest, gene expression analysis, and EGFR inhibition assay.

## Results

### Chemistry

**Fig. 3:**
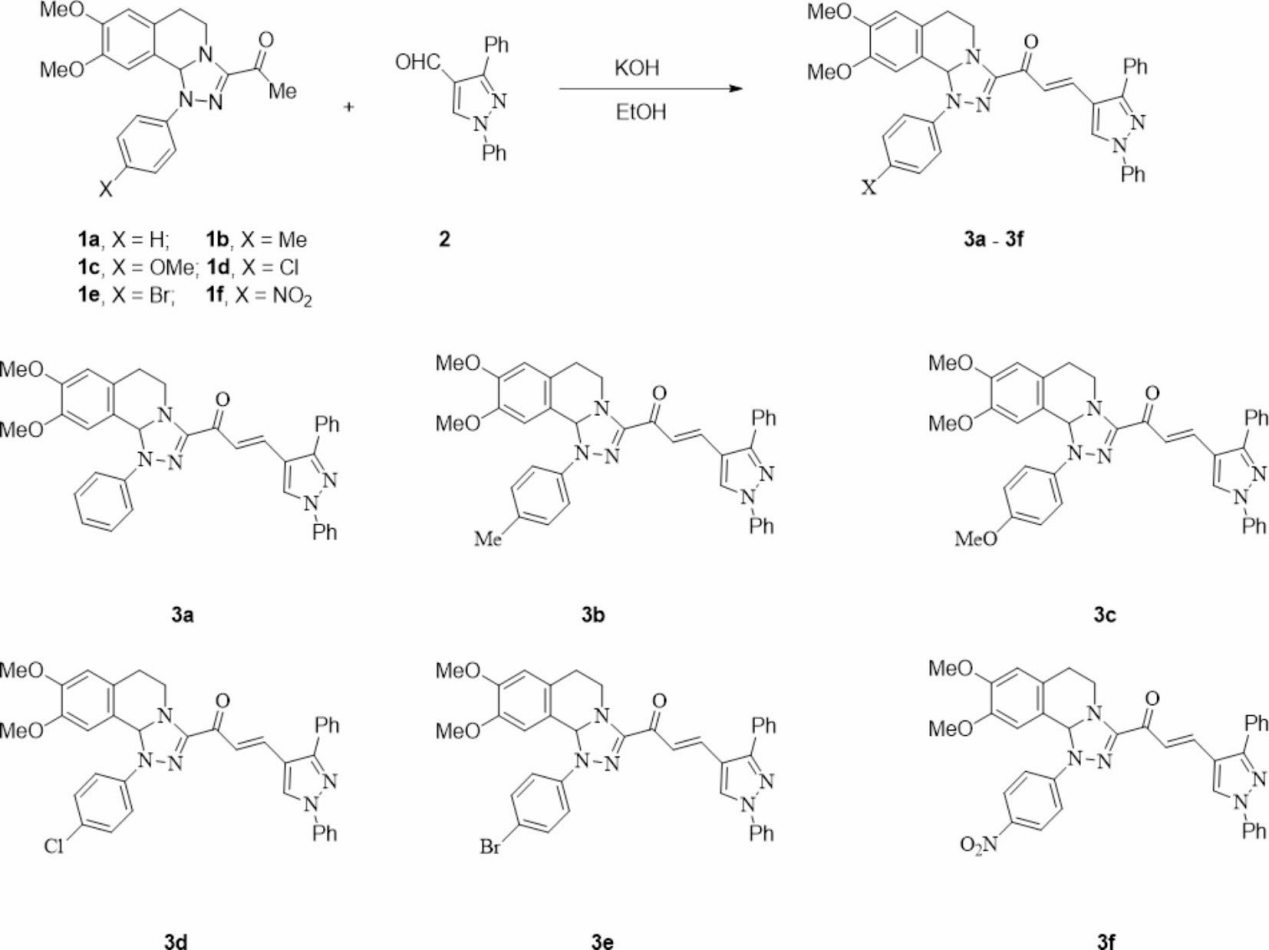
The newly synthesized [1,2,4]triazolo[3,4-*a*]isoquinolin-3-yl)-3-(1,3-diphenyl-1*H*-pyrazol-4-yl)prop-2-en-1-ones compounds (**3a**–**3f**).

The chalcones incorporating [l,2,4]triazolo[3,4-*a*]isoquinoline and 1,3-diphenyl-1*H*-pyrazol scaffolds **3a**–**f** were prepared in moderate to excellent yields *via* the Claisen–Schmidt condensation reaction of 3-acetyl tetrahydro-[1,2,4]triazolo[3,4-*a*]isoquinoline **1** with the mole equivalents of substituted 1-phenyl-pyrazole-4-carbaldehydes **2** in ethanol in the presence of potassium hydroxide solution (20%) (Fig. 3). The structures of the formed products were verified based on spectral analyses.

### Computational chemistry

#### Molecular docking

Modelling studies were used to visualize the binding model of six derivatives within the Erlotinib binding site in the EGFR enzyme, which is upregulated in most tumor cells to defend itself for survival. The molecular docking studies were done using MOE 2009.10 software, and the X-ray structure of the protein was obtained from the protein data bank (PDB ID: 1M17)^64^. This well-characterized crystal structure was utilized to carry out meticulous computational verifications for the discovery of new EGFR inhibitors of medical significance using high structural quality, availability of experimental data, and alignment with previous studies. The results of the molecular docking studies are illustrated in Table 1.

**Table 1:** Binding energy scores (kcal/mol) and interactions of the synthesized compounds (**3a**–**3f**) against the amino acid residues of the active site of EGFR protein (PDB ID: 1M17).

Compd	Docking score (kcal/mol)	Ligand	Receptor	Type of interaction	Distance (Å)	E (kcal/mol)
**3a**	− 8.0205	5-ring	CG1 VAL 702	pi-H	4.05	− 1.0
		6-ring	CD LYS 721	pi-H	3.79	− 0.8
		6-ring	NZ LYS 721	pi-cation	4.53	− 0.5
**3b**	− 7.9611	5-ring	CG1 VAL 702	pi-H	4.14	− 0.8
		5-ring	CG2 VAL 702	pi-H	4.14	− 0.5
		6-ring	NZ LYS 721	pi-cation	3.69	− 0.6
**3c**	− 8.3436	5-ring	CG1 VAL 702	pi-H	4.11	− 0.8
		6-ring	CD LYS 721	pi-H	3.82	− 0.6
		6-ring	NZ LYS 721	pi-cation	4.51	− 0.6
**3d**	− 8.2250	6-ring	CD LYS 721	pi-H	3.83	− 0.8
		6-ring	NZ LYS 721	pi-cation	4.40	− 1.2
		5-ring	CD ARG 817	pi-H	4.34	− 0.8
**3e**	− 8.4115	6-ring	CD LYS 721	pi-H	3.73	− 0.7
		6-ring	NZ LYS 721	pi-cation	4.21	− 0.8
		6-ring	NZ LYS 721	pi-cation	4.68	− 1.7
**3f**	− 9.0861	O 45	SD MET 742	H-donor	3.33	− 0.7
		6-ring	CD LYS 721	pi-H	3.66	− 0.8
		6-ring	NZ LYS 721	pi-cation	4.31	− 0.6
		6-ring	NZ LYS 721	pi-cation	4.65	− 1.8

**Fig. 4:**
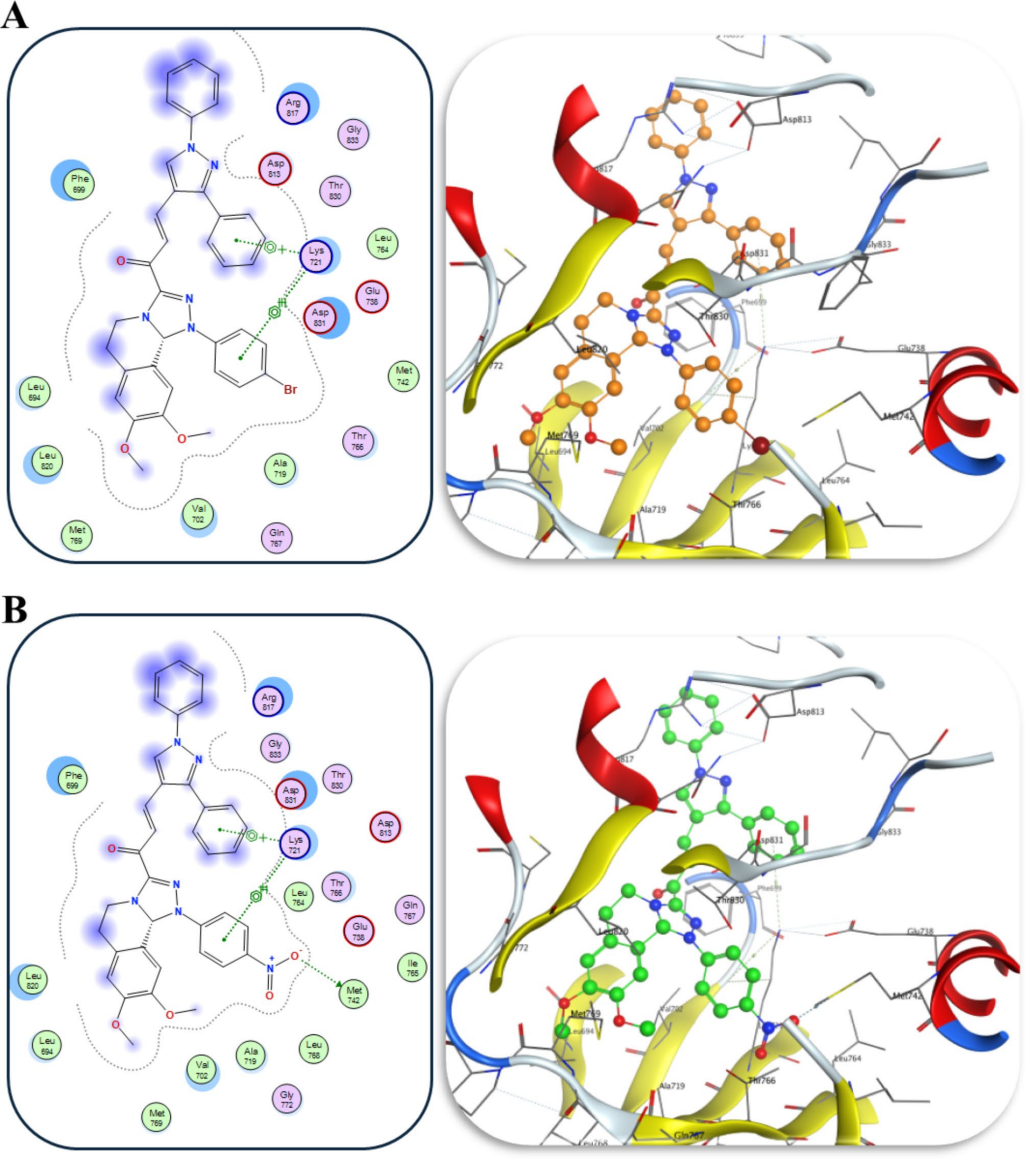
Chalcones (**3e**, **A**; **3f**, **B**) interactions (2D, left panel; 3D, right panel) with EGFR tyrosine kinase ATP binding site (PDB ID: 1M17).

The results showed that our synthesized chalcone derivatives were fitted well within the binding pocket of EGFR, with significant binding scores ranging from − 9.086 to − 7.9611 kcal/mol (Table 1; Fig. 4 and Fig. S1). They interacted with the amino acids surrounding the co-crystalized ligand, erlotinib, as illustrated in Fig. S2. Chalcone **3f** has the highest affinity of binding to the active site of 1M17, with binding score of − 9.086 kcal/mol through four interactions (Fig. 4A) achieved by this drug against EGFR protein. Where the **3f** interacted with H-bond with the amino acid Met742 with bond distance of 3.33 Å and binding energy of − 0.7 kcal/mol. In addition to one pi-H interaction with Lys721 with bond distance of 3.66 Å and binding energy of − 0.8 kcal/mole, and two pi-cation interactions with Lys721 (NZ) with bond distances of 4.31 and 4.65 Å and binding energy − 0.6 and − 1.8 kcal/mol, respectively.

Compound **3e** showed the second-highest binding score of − 8.412 kcal/mol through three interactions (Fig. 4B), where **3e** interacted by pi-H interaction with Lys721 with bond distance of 3.73 Å and binding energy of − 0.7 kcal/mol and two pi-cation interactions with Lys721 with bond distance of 4.21 and 4.68 Å and binding energy of − 0.8 and − 1.7 kcal/mol, respectively. Compound **3c** (Fig. S1A) revealed a very good binding affinity toward EGFR pocket with binding score of − 8.344 kcal/mol, where **3c** showed three types of interactions including two pi-H interactions with Val702 and Lys721 with bond distances of 4.11 and 3.82 Å, respectively, and bond energies of − 0.8 and − 0.6 kcal/mol, respectively, and one pi-cation interaction with Lys721 with distance of 4.51 Å and binding energy of − 0.6 kcal/mol. Chalcone **3d** showed a good binding score of − 8.225 kcal/mol through three non-covalent interactions (Fig. S1B), two pi-H interactions with Lys721 and Arg817, bond distances of 3.83 and 4.34 Å, respectively, and bond energies of − 0.8 and − 0.8 kcal/mol, respectively, and one pi-cation interaction with Lys721 with bond distance of 4.40 Å and bond energy of − 1.2 kcal/mol. Also, chalcone **3a** showed a good binding score of − 8.021 kcal/mol with three non-covalent interactions (Fig. S1C), including two pi-H interactions with Val702 and Lys721 showing distances of 4.05 and 3.79 Å, respectively, and bond energies of − 1.0 and − 0.8 kcal/mol, respectively, and one pi-cation interaction with Lys721, distance of 4.53 Å and bond energy of − 0.5 kcal/mol. Finally, compound **3b** revealed the least binding energy score among the investigated compounds, − 7.961 kcal/mol, with three non-covalent bonds (Fig. S1D) including two pi-H interaction with CG1 and CG2 of Val702 with bond distance of 4.14 Å and bond energy of − 0.8 and − 0.5 kcal/mol, respectively, and one pi-cation interaction with Lys721, bond length of 3.69 Å and bond energy of − 0.6 kcal/mol.

### Biological activity evaluation

#### Cytotoxic activity

The final six structurally confirmed based quinazoline derivatives were biologically evaluated for their in vitro antiproliferative activity via MTT assay, against a panel of five human cancer cell lines namely, colorectal carcinoma (HCT-116) (HT29), non-small cell lung cancer (A549), prostatic adenocarcinoma (PC3), breast cancer (MCF-7) and normal lung (WI-38) cell lines. Four commercially available drugs, 5-fluorouracil (5-FU) and EGFR inhibitors (lapatinib, Erlotinib, and Gefitinib), were used as positive controls while the left untreated cells served as the negative control. The results were expressed as half-growth viability concentration (IC_50_) values and summarized in Table 2.

**Table 2:** In vitro anticancer screening of compounds (**3a-3f**) against 5 cancer cell lines and normal cell line compared to standard drugs.

Compound	IC_50_ (µM)					
	HCT-116	HT-29	A549	PC3	MCF-7	WI-38
**3a**	8.15 ± 0.54	16.22 ± 0.57	1.98 ± 0.5	3.14 ± 1.76	8.07 ± 0.1	890.44 ± 0.13
**3b**	9.41 ± 0.47	5.05 ± 0.42	2.45 ± 0.68	6.61 ± 1.63	10.02 ± 0.23	669.88 ± 0.26
**3c**	5.4 ± 0.73	4.69 ± 0.55	5.26 ± 0.49	3.22 ± 1.37	4.07 ± 0.38	451.08 ± 0.11
**3d**	14.07 ± 0.66	15.01 ± 0.58	4.68 ± 0.41	5.05 ± 1.43	13.47 ± 0.29	468.92 ± 0.17
**3e**	14.76 ± 0.31	4.2 ± 0.38	2.3 ± 0.11	2.72 ± 1.36	4.21 ± 0.41	1731.93 ± 0.21
**3f**	4.61 ± 0.28	2.41 ± 0.41	1.15 ± 0.38	22.16 ± 0.18	5.53 ± 0.33	1578.05 ± 0.23
**5-FU**	171.48 ± 0.23	203.55 ± 0.48	66.24 ± 0.36	178.2 ± 0.34	160.31 ± 0.47	210.69 ± 0.19
**Lapatinib**	5.92 ± 0.45	8.23 ± 0.33	9.66 ± 0.44	13.72 ± 0.89	5.02 ± 1.1	1239.44 ± 0.18
**Erlotinib**	12.31 ± 0.38	9.50 ± 0.46	7.32 ± 0.52	14.41 ± 0.78	3.11 ± 0.14	1431.64 ± 0.22
**Gefitinib**	9.52 ± 0.59	14.72 ± 0.71	12.46 ± 0.19	18.36 ± 0.21	21.73 ± 0.12	895.72 ± 0.24

IC_50_ values = mean ± SD of three independent determinations.

All the compounds displayed good cytotoxic activity against all selected carcinoma cell lines compared with 5-FU, as illustrated in Table 2. With the exception of **3d** and **3e**, the compounds’ IC_50_ values against HCT-116 cancer cells varied from 4.61 to 14.76 µM, which were lower than those of the reference drugs. The lung cancer line (A549) showed a significantly low surviving fraction toward all compounds with IC_50_ values (1.15–5.26 µM), which was more potent than the reference drugs. All compounds recorded a moderate cytotoxic activity with IC_50_ values ranging from 2.72 to 22.16 µM against the prostate carcinoma cell line (PC3). Further, a decrease in the cytotoxic activity on HT-29 was noticed with IC_50_ values ranging from 2.41 to 16.22 µM. Meanwhile, for MCF-7 breast cancer cells, compounds **3e**, **3f**, and **3c** showed pomising IC_50_ values of 4.21, 5.53, and 4.07 µM, respectively. Compound **3b** scored a good IC_50_ value of 10.02 µM on breast cancer (MCF-7), however, compounds **3d** and **3a** had moderate inhibition on MCF-7 cells.

**Fig. 5:**
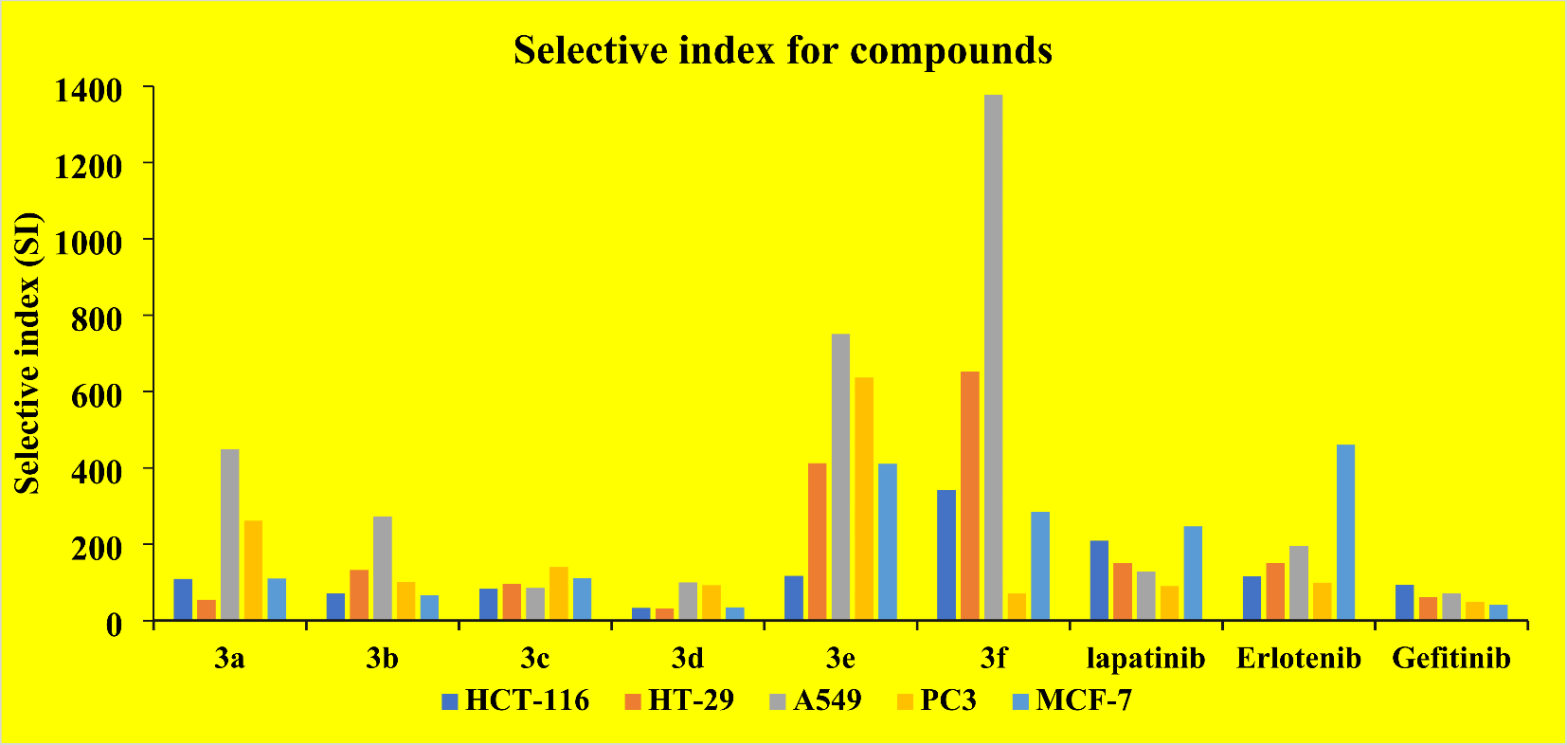
Comparison between the selective index (SI) of each chalcone and the reference drugs against mammalian cancer lines; HCT-116 and HT-29: colorectal adenocarcinoma, A549: non-small cell lung cancer cells, PC-3: prostatic adenocarcinoma, MCF-7: breast cancer and WI-39: normal lung cell line. SI = IC_50_ for normal cells/IC_50_ for cancer cells.

Comparing the resultant activities of the compounds with 5-fluorouracil 5-FU) from a selectivity index (SI) perspective (Fig. 5), where the selectivity index for an anticancer compound is a measure of its relative cytotoxicity towards cancer cells compared to normal cells, the whole series showed an excellent selectivity index for A549 lung cancer cells. While chalcone **3f** achieved the highest SI on A549 than PC3, it is seven times safer on lung cancer cells than Erlotinib. Compound **3c** displayed a very good selectivity index toward A549, 5.6 times safer on lung cancer cells than Erlotinib. Besides, chalcone **3e** was 3.8 times safer than Erlotinib. To investigate the mechanism of action suggested by molecular docking studies and the cytotoxic screening, the series were further evaluated for their inhibitory activity of EGFR.

**Structural-activity relationship (SAR)**.

**Fig. 6:**
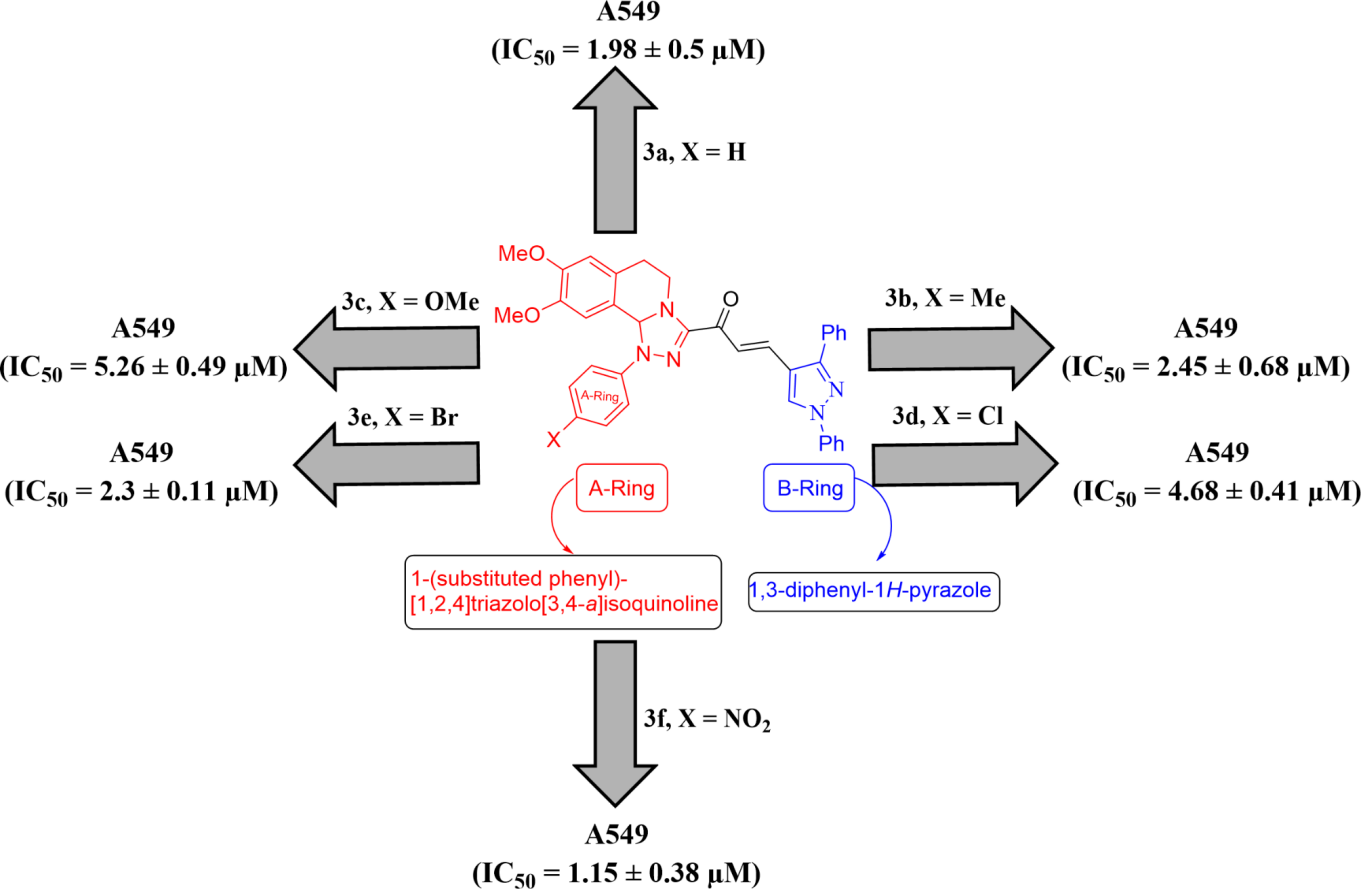
Structural-activity relationship of compounds **3a–3f**.

The structure can be represented as *α*,*β*-unsaturated enone group attached to A-ring (the substituted aryl group attached to [1,2,4]triazolo[3,4-*a*]isoquinoline group) and B-ring(1,3-diphenyl-1*H*-pyrazole) (Fig. 6). The type of the substituents had a substantial impact on the cytotoxic actions. It is noteworthy that the unsubstituted **3a** and derivatives with *electron-donating groups  ***3b** and **3c** were more potent than *electron-withdrawing groups* containing derivatives (**3d**, **3e**, and **3f**) against the tested cancer (HCT-116 and PC3) cells, except for **3b** and **3e** in the case of PC3 cells. On the other hand, the compounds with electron-withdrawing groups (**3e** and **3f**) are more potent than the unsubstituted **3a** and compounds containing electron-donating groups (**3b** and **3c**) against the cell lines (HT-29, A549, and MCF-7). Notably, the chloro-substituted derivative **3d** revealed the lowest cytotoxic potential among the synthesized derivatives against the examined cell lines.

#### Compounds 3e and 3f induce apoptosis and necrosis as well as cell cycle arrest

**Fig. 7:**
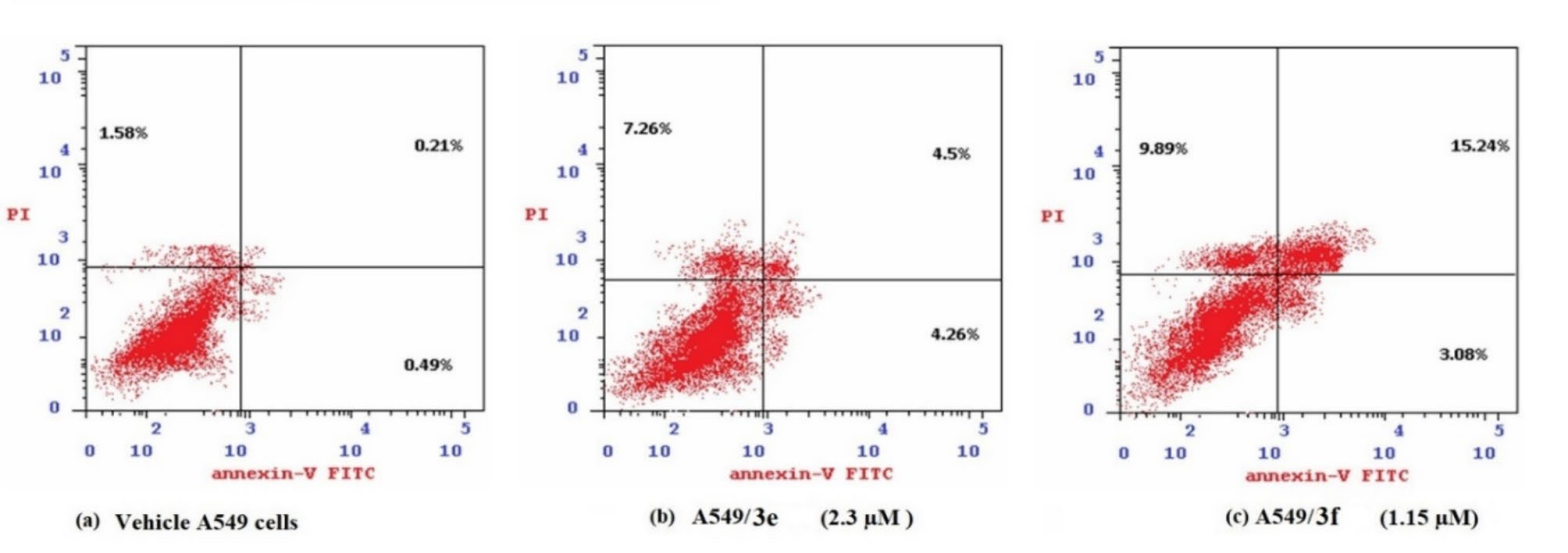
Effect of chalcones **3e** (2.3 µM) and **3f** (1.15 µM) on cell death by flow cytometric analysis of A549 cells after 24 h.

The cell-cycle mechanism is one of the most common modes of action for anticancer agents. The ability of compounds to inhibit cancer cell growth was investigated by inducing apoptosis at various stages and measuring cell cycle arrest^65^. In Fig. 7, the annexin V/PI flow cytometric analysis indicated an increase in the percentage of apoptosis for **3e**-A549 treated cells. The early apoptosis phase was increased from 0.49% to 4.26%, as well as a significant increase in the late apoptosis phase from 0.21% to 4.5% was observed. Thus, there was an overall increase in the percentage of total apoptosis from 0.7% to 8.76%. Moreover, the percentage of cells in the necrotic phase was increased from 1.58% to 7.26%. In addition, **3f**-treated A549 treated cells revealed a significant elevation in the percentage of early apoptotic cells from 0.49% to 3.08%, and an elevation in the late apoptotic cells from 0.21% to 15.24%. So, the increase in the total apoptotic percentage was from 0.7% to 18.32%. In the late stage of apoptosis, caspases-dependent and -independent pathways lead to DNA fragmentation and nuclear condensation. Furthermore, an elevation in the percentage of the cells in the necrotic phase from 1.58% to 9.89% was observed.

As shown in Table 3, compounds **3e** and **3f** significantly increased apoptotic death of A549 lung cells, reaching 8.76% and 18.32%, respectively, compared to their control (0.7%). Thus, **3f** was more active in cell death induction than **3e**.

**Table 3:** Apoptotic analysis of A549 lung cancer cells after 24 h of treatment with the selected chalcones.

Sample	Apoptosis			Necrosis (%)
	Early (%)	Late (%)	Total (%)	
**3e**/A549	4.26	4.5	8.76	7.26
**3f**/A549	3.08	15.24	18.32	9.89
A549 vehicle cells	0.49	0.21	0.70	1.58

**Fig. 8:**
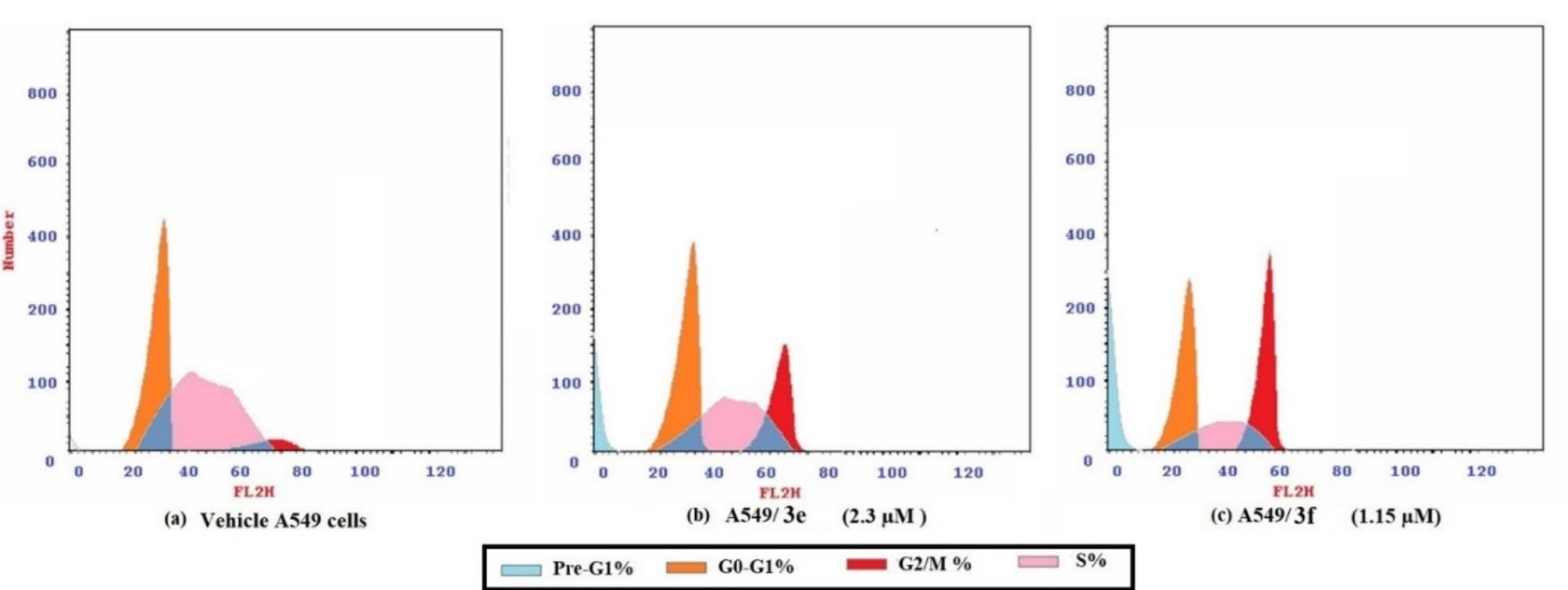
Graphical representation of the cell cycle analysis of chalcones **3e** and **3f** relatives to their control in A549 lung cancer cells.

As a result, the cell distribution of A549 cells treated with the chalcones **3e** and **3f** in each phase of the cell cycle was determined using a cell cycle assay. According to the data obtained in Fig. 8 and Table 4, 3e and 3f diminished the cell population in the G0/G1 phase from 46.75% to 41.33% and 33.74%, respectively. In addition, the percentage of cells in the S phase decreased from the initial 44.26% to a final 35.93% and 26.42% for **3e** and **3f**-treated cells, respectively. On the other hand, a substantial increase in cell proportions was illustrated at the G2/M phase from 8.99% in the control cells to 22.74% and 39.84% for **3e** and **3f**-treated cells, respectively. Also, a significant increase in the cell populations at the pre-G1 phase from 2.28% in control cells to 16.02% and 28.21% for **3e** and **3f**-treated cells, respectively. Therefore, compounds **3e** and **3f** induced cell growth arrest at the G2/M and pre-G1 phases when compared to the untreated control cells.

**Table 4:** Cell cycle analysis of A549 lung cancer cells after 24 h treatment of chalcones **3e** and **3f** relative to their A549 control cells.

Sample	DNA content			
	Pre-G1%	G0–G1%	S%	G2/M%
**3e**/A549	16.02	41.33	35.93	22.74
**3f**/A549	28.21	33.74	26.42	39.84
A549 vehicle cells	2.28	46.75	44.26	8.99

Collectively, chalcones **3e** and **3f** have proven their potential through induction of apoptosis and some degree of necrosis as measured by flow cytometry. In addition, analysis of the cell cycle under the effect of IC_50_ of both compounds has revealed a cell cycle arrest at the G2/M and pre-G1 phases.

#### Compounds 3e and 3f up-regulate apoptotic genes and down-regulate antiapoptotic genes

**Fig. 9:**
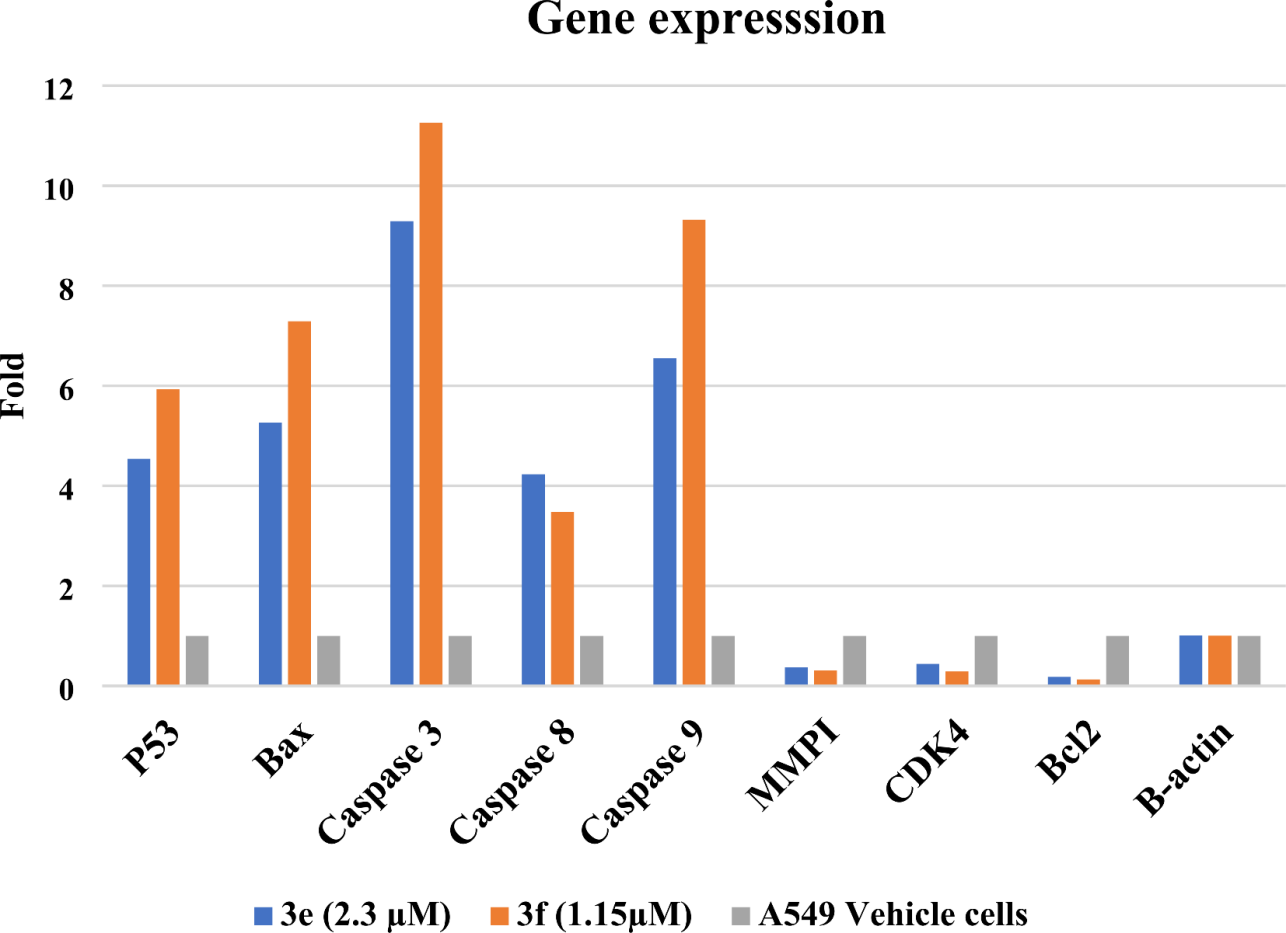
Gene expression analysis of A549 lung cancer cells treated by **3e** and **3f** chalcones and vehicle cells.

In Fig. 9, the data revealed that chalcones **3e** and **3f** significantly upregulated the five tested pro-apoptotic markers (p53, Bax, caspases 3, 8, and 9) with the highest value (fold change = 9.29, 11.26) observed in caspase-3 (executive protein of apoptosis), respectively. Contrastingly, there was a significant downregulation in the three tested antiapoptotic markers (MMP1, CDK4, and Bcl2) with the most decline (fold change = 0.44, 0.29) for (**3e** and **3f**-A549 treated cells) being seen in CDK4, respectively.

#### EGFR inhibition assay

Data presented in Table 5 reflected the inhibitory activity of six compounds on the total EGFR. Chalcones **3e** and **3f** exhibited a stronger inhibitory activity with IC_50_ values of 0.031 and 0.023 µM, respectively, compared to standard chemotherapeutic drugs namely: Lapatinib, Erlotinib, and Gefitinib with IC_50_ values of 0.044, 0.046, and 0.095 µM, respectively. Meanwhile, compounds **3c** and **3d** showed a considerable activity better than erlotinib but were less potent than the other two chemotherapeutic agents.

**Table 5:** Total EGFR inhibitory activity of the synthesized compounds (**3a**–**3f**) compared to Lapatinib, Gefitinib, and Erlotinib as reference standards.

Compound	EGFR IC_50_ (µM)
**3a**	1.623 ± 0.23
**3b**	1.885 ± 0.37
**3c**	0.059 ± 0.16
**3d**	0.066 ± 0.24
**3e**	0.031 ± 0.31
**3f**	0.023 ± 0.29
Lapatinib	0.044 ± 0.32
Gefitinib	0.046 ± 0.28
Erlotinib	0.095 ± 0.34

**Fig. 10:**
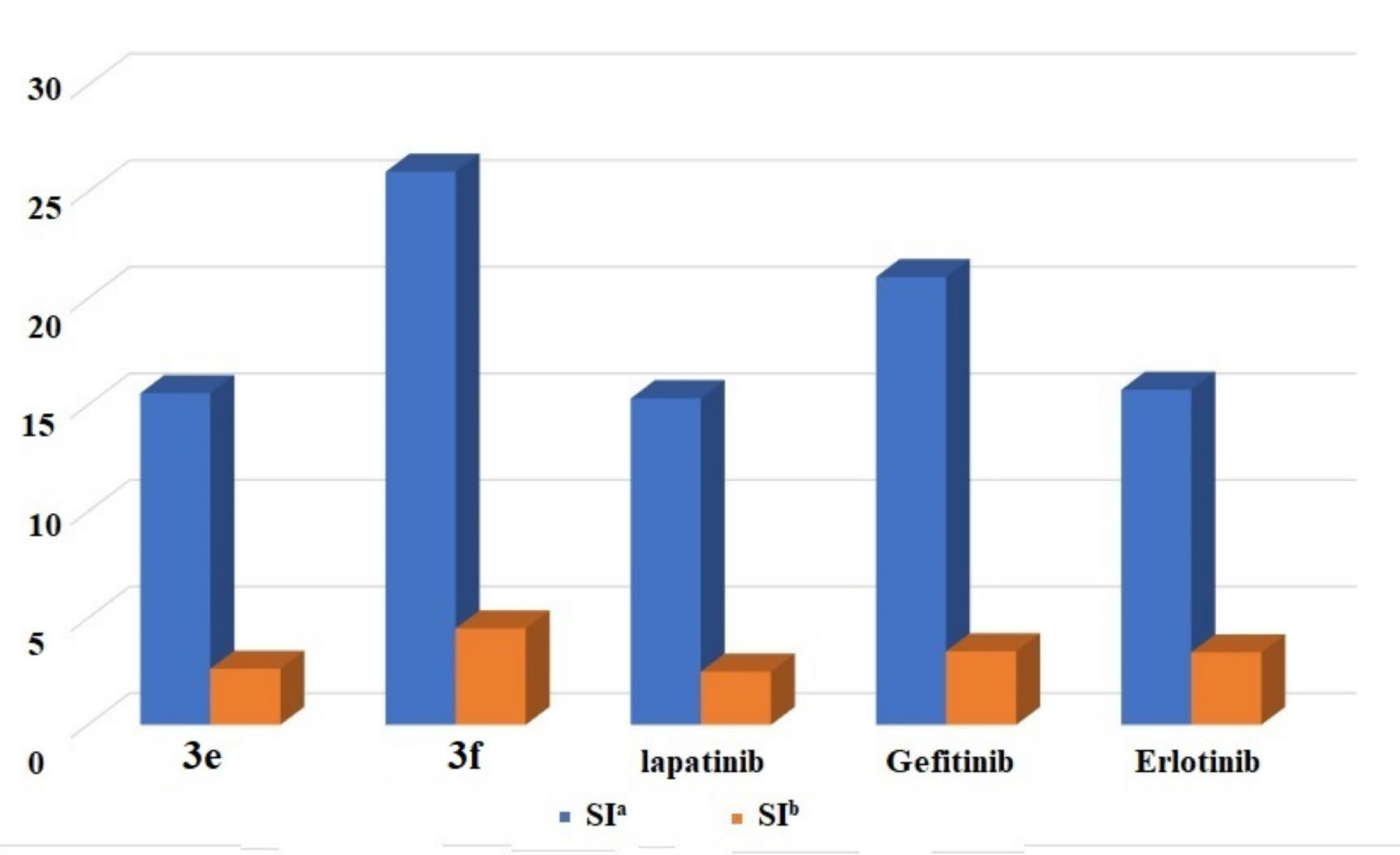
Selectivity index of the tested compounds **3e** and **3f** relative to the drugs for EGFR proteins: EGFR^L858R^, EGFR^T790M^, and EGFR^wt^. Selectively index (SI^a^) = EGFR^WT^ IC_50_/ EGFR^L858R^ IC_50_ value, Selectively index (SI^b^ ) = EGFR^WT^ IC_50_/ EGFR^T790M^ IC_50_ value.

The most potent inhibitory chalcones **3e** and **3f** were tested further for their inhibitory activity against two mutated EGFR proteins. The three investigated receptors are EGFR^wt^ (wild-type EGFR), EGFR^T790M^ (EGFR with T790M mutation), and EGFR^L858R^ (EGFR with L858R mutation). Both compounds achieved stronger inhibitory activity on the mutations compared to the three standard chemotherapeutics (Table 6). Chalcone **3e** showed significant inhibitory activity against EGFR^wt^ with a moderate selectivity index toward EGFR^L858R^. While chalcone **3f** showed the highest inhibitory activity against EGFR^L858R^ and EGFR^T790M^, with the lowest IC_50_ values of 34.78 µM and 199.53 µM, respectively. Chalcone **3f** also exhibited the highest selectivity index for EGFR^L858R^, and EGFR^T790M^ with SI = 25.95 and 4.52, respectively (Fig. 10).

**Table 6:** Inhibitory activities of chalcones **3e** and **3f** against EGFR-TKs compared to Lapatinib, Gefitinib, and Erlotinib as reference standards.

Compound	IC_50_ (µM)			SI^a^	SI^b^
	EGFR^L858R^	EGFR^T790M^	EGFR^wt^		
**3e**	46.02 ± 0.08	272.74 ± 0.02	716.43 ± 0.05	15.56	2.63
**3f**	34.78 ± 0.05	199.53 ± 0.07	902.76 ± 0.17	25.95	4.52
**Lapatinib**	62.15 ± 0.09	379.8 ± 0.13	952.4 ± 0.08	15.32	2.50
**Gefitinib**	79.86 ± 0.12	485.99 ± 0.10	1678.46 ± 0.16	21.01	3.45
**Erlotinib**	91.03 ± 0.11	419.89 ± 0.05	1431.22 ± 0.04	15.72	3.40

Selectivity Index (SI^a^) = EGFR^WT^ IC_50_/ EGFR^L858R^ IC_50_ value, Selectivity Index (SI^b^) = EGFR^WT^ IC_50_/ EGFR^T790M^ IC_50_ value.

## Discussion

Medicinal chemists have proven various biological activities by adding different active groups to the quinoline molecule using various synthesis procedures [31]. It is now possible to design potent drugs by targeting receptors’ mutations using computer-aided drug design tools. The current study design is based on the aforementioned basic information about EGFR inhibitors and their role in cancer therapy. In order to overcome the resistance produced by prior chemotherapeutics, a series of very effective triazolo[3,4-a]isoquinoline derivatives have been synthesized and submitted to in silico and in vitro biological assessments.

Compounds **3e** and **3f** exhibited significant inhibitory activities with IC_50_ values of 0.031 µM and 0.023 µM, respectively, which are more potent than the reference chemotherapeutic drugs. Chalcone **3e** is 1.42-fold more efficient in inhibiting EGFR transmembrane protein than lapatinib while chalcone **3f** is 1.91-fold more effective than Lapatinib. As previously mentioned, the increased potency of chalcone **3e** (Fig. 4B) could be related to the presence of pi-H interaction with Lys721 and two pi-cation interactions with Lys721.

Chalcone **3f**, which displayed the most significant inhibitory activity, is more potent than erlotinib, gefitinib, and lapatinib. This enhanced potency could be attributed to the presence of H-bond with the amino acid Met742, one pi-H interaction with Lys721, and two pi-cation interactions with Lys721 (Fig. 4A). Furthermore, the significant activity of chalcones **3e** and **3f** against EGFR^L858R^ and EGFR^T790M^ and their corresponding IC_50_ values (IC_50_ values of **3e**: 46.02 ± 0.08, 272.74 ± 0.02 µM) (IC_50_ values of **3f**: 34.78 ± 0.05, 199.53 ± 0.07 µM) were supported by their good docking score (− 8.412 and − 9.086 kcal/mol, respectively), additionally, their excellent superimposition on the ligand (erlotinib) in the active site.

To understand the mechanism by which these compounds exert their anticancer activities, different assays were performed including MTT, cell cycle analysis, apoptosis, and molecular gene expressions of 5 pro-apoptotic genes (P53, Bax, Caspase 3, Caspase 8, and Caspase 9) and 3 anti-apoptotic genes (MMP1, CDK4, and Bcl2). MTT assay was carried out to determine the cytotoxic effects of new anilino-quinazoline derivatives on five cancer cells (HCT116, HT29, A549, PC3, and MCF-7 cell lines). 5-Fluorouracil was used as a standard anticancer chemotherapeutic reference, as reported in several studies dealing with the anticancer activities of anilino-quinazoline^49^, and Erlotinib, Lapatinib, and Gefitinib were also chosen based on their inhibitory activity against EGFR. The selection of 5-fluorouracil is based on its mechanism of action which has been attributed to apoptosis induction in cancer cells. Although all the series showed a good cytotoxic effect on the five cell lines, chalcones **3e** and **3f** showed a promising antiproliferative potential on A549 cells with IC_50_ values of 2.3 ± 0.11 µM, and1.15 ± 0.38 µM, respectively, when compared with Erlotinib, Lapatinib, and Gefitinib (IC_50_ = 7.32 ± 0.52 µM, 9.66 ± 0.44 µM, and 12.46 ± 0.19 µM, respectively) (Table 2).

Apoptosis is a regulated and physiological mechanism to cell death. Cancer is generally caused by a disruption in the apoptotic pathway. An essential feature of anticancer drug safety is the capacity to generate planned apoptosis rather than uncontrolled necrotic cell death. Thus, most anticancer chemotherapeutic drugs target the induction of apoptosis^66^. Apoptosis resulted in the progressive production of many biochemical markers. Apoptotic proteins being released, caspase activation, and phosphatidylserine externalization are some of these markers^67^. A cytofluorimetric analysis was used to distinguish between cell death mechanisms. using Annexin V-FITC assay, where propidium iodide (PI) stains dead cells’ DNA. While Annexin-V binds to phosphatidylserine (PS), which is exclusively expressed on the surface of apoptotic cells and fluoresces green after engaging with the fluorochrome-labelled Annexin-V^68^. Since chalcones **3e** and **3f** showed promising anticancer activity against A549 cells, cells were treated with chalcones **3e** and **3f** at their IC_50_ values (2.3 and 1.15 µM), respectively, for 24 h. The quantitative assessment of apoptosis provided by Annexin V-FITC/PI labelling with flow cytometry (Fig. 7) revealed that the A549 cells treated with chalcones **3e** and **3f** showed a remarkable induction of total apoptosis as well as necrosis in comparison to untreated cells (*P* < 0.05). The data in Fig. 8 revealed that chalcone **3e** and **3f** arrested the cell cycle of the A549 cells at G2/M and pre-G1 phases. Takac et al.^69^ concluded that chalcones suppressed cancer cell multiplication by interrupting the cell cycle. The anticancer properties of chalcone analogues might be correlated to their structural similarity to 5-FU. In this case, 5-FU was discovered to block thymidylate synthase, which prevents DNA synthesis and induces apoptosis. Thus, the apoptotic alterations seen in the cells of the current investigation may be related to the suppression of DNA synthesis and consequent cell cycle arrest^70^.

The gene expression level of the following genes - five pro-apoptotic genes (P53, Bax, Caspase 3, Caspase 8, and Caspase 9) and three anti-apoptotic genes (MMPI, CDK4, and Bcl2) - were investigated using specific primers (experimental section) for each gene in **3e **and **3f**-treated A549 lung carcinoma cell line, compared to the housekeeping gene (beta-actin). Chalcones **3e** and **3f** have been shown to induce significant increase in the P53/Bcl-2 ratio (*P* < 0.05) when compared to the A549 vehicle control cells.

Caspases are accountable for the deliberate disassembly of the cell into apoptotic bodies during apoptosis. Caspases 3, 8, and 9 proteins are situated at pivotal junctions in apoptosis pathways^71^. Compounds **3e** and **3f** exhibited a potent upregulated effect on the expression level of their respective genes (9.29, 4.23, and 6.55) and (11.26, 3.48, and 9.32) folds, respectively. Contrastingly, chalcones **3e** and **3f** significantly downregulated the MMP1 (fold change = 0.37, 0.31), CDK4 (fold change = 0.44, 0.29), and Bcl-2 (fold change = 0.18, 0.13) genes as shown in Fig. 9. The ratio between Bax and Bcl-2 levels is crucial for the condemnatory mitochondrial apoptosis pathway since the Bax gene induces apoptosis while Bcl-2 suppresses apoptosis. The upregulation of pro-apoptotic genes (P53, Bax, Caspase 3, and Caspase 9) and the downregulation of the anti-apoptotic genes (MMP1, CDK4, and Bcl2) after the treatment with chalcones **3e** and **3f** implicit the inhibition of cancer cells through the intrinsic pathway of apoptosis, which was a positive correlation between the cytotoxic efficacy of the compounds and apoptosis in A549 cells.

These findings support the idea that the intrinsic (mitochondrial) apoptotic pathway is involved in the anticancer activity of these new chalcone anilino-quinazoline derivatives. Chalcones have been shown to activate Bax, inhibit Bcl-2, and activate caspase 9, which is consistent with our findings. According to Chen et al.^72^, a chalcone (lonchocarpin) induced apoptosis through regulating Bax, caspase 9, and caspase 3 expression. Furthermore, recent research has demonstrated that chalcones act as an apoptotic regulator in human breast, lung, and hepatic cancer cells, and inhibiting cancer cell metastasis^73^. Caspase-independent pathways are mediated in the late stage of apoptosis, leading to DNA fragmentation and nuclear condensation.

## Conclusion

A series of novel Chalones was synthesized and confirmed by spectral and NMR methods. Then, they were screened for their cytotoxic activity against five mammalian cancer cell lines and one normal cell line. All the compounds showed wide anticancer activity on the cancer cell lines, and the selectivity index was calculated for each compound. A549 lung cancer cells were the most responsive cells among the investigated cells toward our novel compounds. The IC_50_ results on A549 cells were promising compared to the reference drugs. Chalcones **3e** and **3f** showed inhibition of total EGFR with IC_50_ values of 0.031 µM and 0.023 µM, respectively. Notably, chalcones **3e** and **3f** were found to be the most potent derivatives against the two EGFR mutations: EGFR^L858R^ and EGFR^T790M^, and wild-type EGFR: EGFR^wt^. Chalcones **3e** and **3f** were found to be selective against EGFR^L858R^ mutation. In addition, the molecular docking results confirmed that the binding patterns of chalcones **3e** and **3f** were consistent with their EGFR-TK inhibitory activity. The most potent chalcone **3f** induced a significant elevation in the late apoptosis phase from 0.21% to 15.24% in the A549 cell line as shown by Annexin V-FITC/PI assay. Chalcone **3f** is more potent than chalcone **3e** by 2.09-fold, leading cells to apoptosis in general and in late apoptosis specifically by 3.39% more than chalcone **3e**. This evidence was reinforced by an increase in the level of apoptotic caspases (3, 8, and 9) by (9.29, 4.23, and 6.55) fold, respectively. Moreover, the results of cell cycle analysis showed that derivatives **3e** and **3f** arrested the cell cycle proliferation of A549 cancer cells in the G2/M and pre-G1 phases. The present study suggested that the newly synthesized anilino-quinazoline derivatives could inhibit EGFR, subsequently inducing apoptosis in A549 cells through the intrinsic pathway as a possible mechanism of their anticancer activity. These results support the antiproliferative activity of chalcone **3f** and may present this compound as a candidate for further biological evaluation as an anticancer agent.

## Experimental section

### Chemistry

#### General procedure for the synthesis of 1-(tetrahydro-[1,2,4]triazolo[3,4-*a*]isoquinolin-3-yl)-3-(1,3-diphenyl-1*H*-pyrazol-4-yl)prop-2-en-1-ones (**3a**–**f**)

A mixture of the appropriate acetyl derivative **1** (0.351 g, 1 mmol) and the pyrazole-4-carbaldehyde **2** (1 mmol) in ethanol (20 mL) containing 5 mL of KOH (20%) was stirred at r. t. for 5 h. The mixture was then transferred over ice containing HCl. The formed yellow product was then filtered, washed with water, and crystallized from the appropriate solvent to give chalcones **3a-f**.

#### 1-(8,9-Dimethoxy-1-phenyl-1,5,6,10b-tetrahydro-[1,2,4]triazolo[3,4-*a*]isoquinolin-3-yl)-3-(1,3-diphenyl-1*H*-pyrazol-4-yl)prop-2-en-1-one


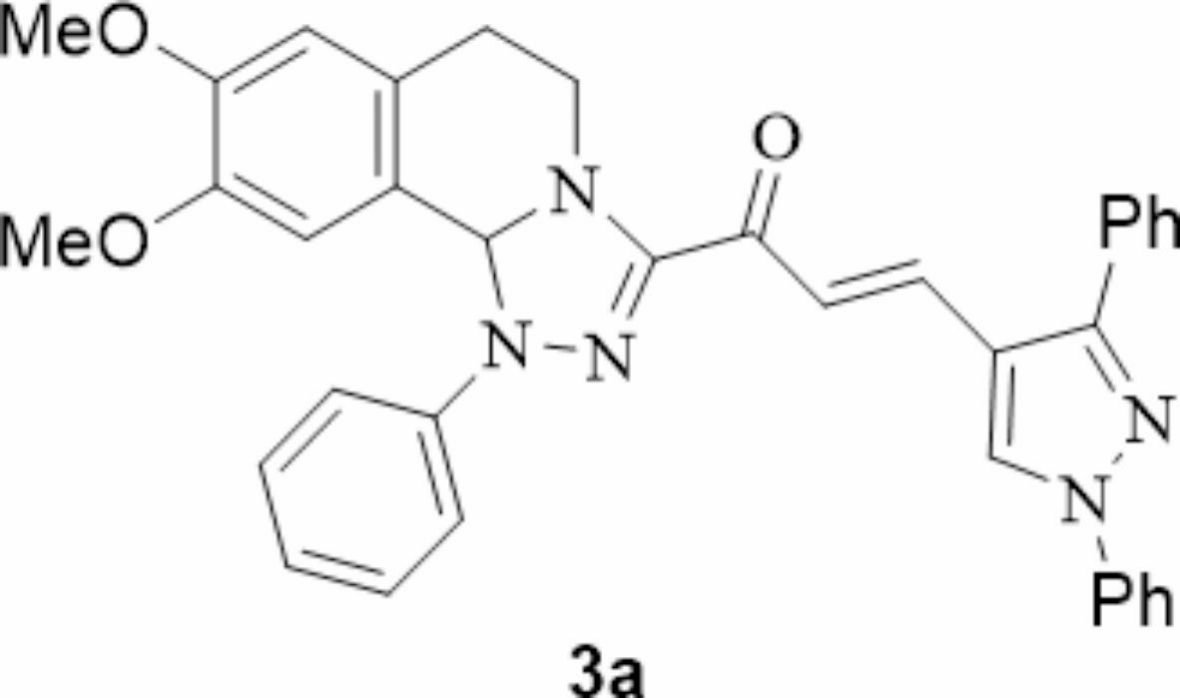


Yield: (88%) as a pale-yellow solid (from ethanol / dioxane); m.p 180–182 °C^74^.

#### 1-(8,9-Dimethoxy-1-(*p*-tolyl)-1,5,6,10b-tetrahydro-[1,2,4]triazolo[3,4-*a*]isoquinolin-3-yl)-3-(1,3-diphenyl-1*H*-pyrazol-4-yl)prop-2-en-1-one


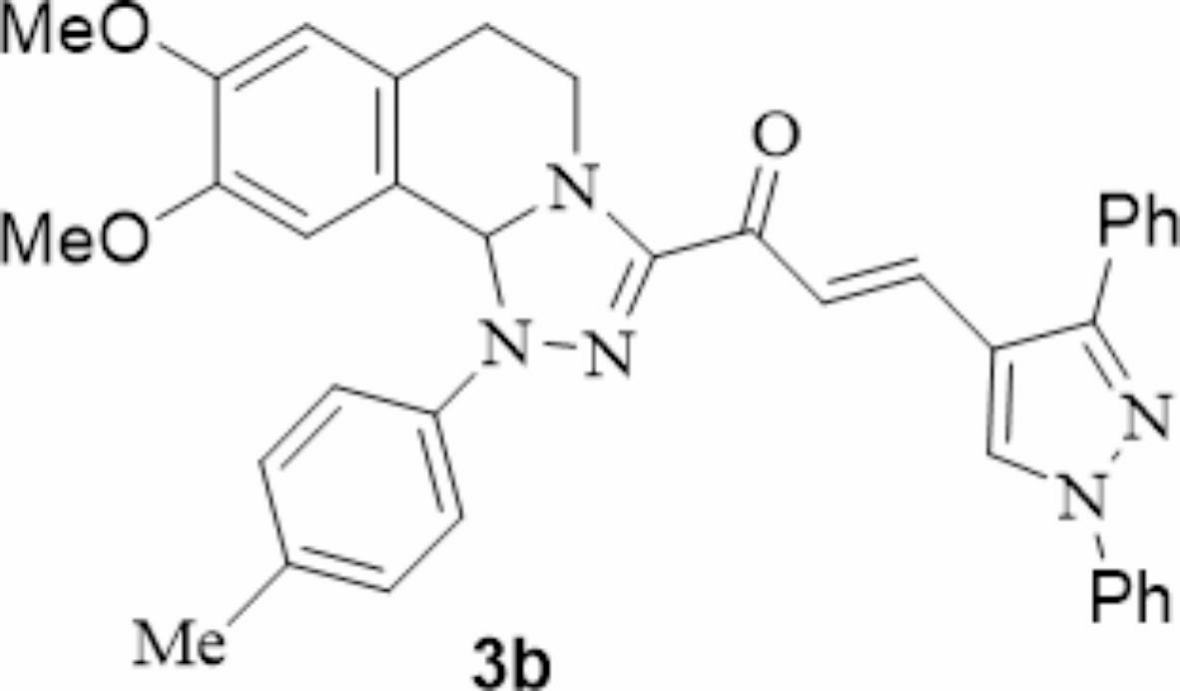


Yield: (85%) as a pale-yellow solid (from ethanol / dioxane); m.p 184–186 °C. IR (KBr, cm^− 1^): 1665 (CO); ^1^H NMR (400 MHz, DMSO-d6): δ, ppm: 2.30 (s, 3 H, Me), 2.62–2.66 (m, 1 H, H6), 3.03–3.33 (m, 1 H, H6), 3.41 (s, 3 H, OMe), 3.71 (s, 3 H, OMe), 3.84–3.87 (m, 1 H, H5), 4.15–4.21 (m, 1 H, H5), 6.63 (s, 1 H, H10b), 6.76 (s, 1 H, H7), 6.88 (s, 1 H, H10), 7.21 (d, 2 H, Ar-H, *J* = 8.36 Hz), 7.28 (d, 2 H, Ar-H, *J* = 8.36 Hz), 7.55–7.66 (m, 10 H, Ar-H and vinyl-H), 7.98 (d, 2 H, Ar-H, *J* = 8.38), 9.33 (s, 1 H, pyrazole-H5);^13^C NMR (100 MHz, DMSO-d6): δ, ppm: 20.8, 27.3, 41.9, 55.8, 55.9, 78.2, 109.4, 112.4, 115.4, 118.1, 119.2, 122.5, 127.4, 127.5, 128.9, 129.0, 129.2, 129.3, 129.4, 130.1, 130.2, 130.5, 132.1, 132.5, 139.4, 141.5, 147.4, 148.9, 149.7, 153.4, 179.3; MS (EI): m/z = 595 (M^+^). Anal. Calcd. for C_37_H_33_N_5_O_3_ (595.70): C, 74.60; H, 5.58; N, 11.76. Found: C, 74.73; H, 5.72; N, 11.82.

#### 1-(8,9-Dimethoxy-1-(4-methoxyphenyl)-1,5,6,10b-tetrahydro-[1,2,4]triazolo[3,4-*a*]isoquinolin-3-yl)-3-(1,3-diphenyl-1*H*-pyrazol-4-yl)prop-2-en-1-one


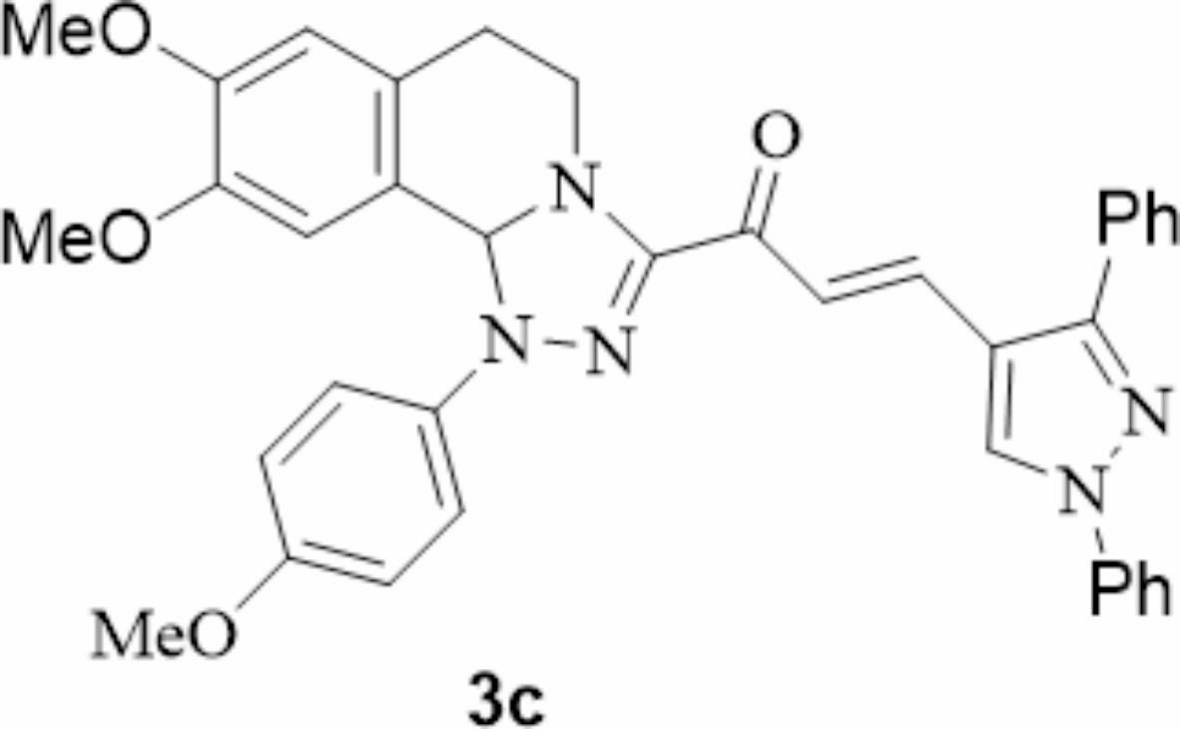


Yield: (82%) as a pale-yellow solid (from ethanol / dioxane); m.p 186–188 °C. IR (KBr, cm^− 1^): 1667 (CO); ^1^H NMR (400 MHz, DMSO-d6): δ, ppm: 2.58–2.62 (m, 1 H, H6), 2.72–2.79 (m, 1 H, H6), 3.40 (s, 3 H, OMe), 3.48–3.58 (m, 1 H, H5), 3.70 (s, 3 H, OMe), 3.76 (s, 3 H, OMe), 4.25–4.28 (m, 1 H, H5), 6.56 (s, 1 H, H10b), 6.73 (s, 1 H, H7), 6.87 (s, 1 H, H10), 7.0 (d, 2 H Ar-H, *J* = 9.04 Hz), 7.33 7.0 (d, 2 H Ar-H, *J* = 9.04 Hz), 7.38–7.44 (m, 1 H, Ar-H), 7.52–7.67 (m, 9 H, Ar-H and vinyl-H), 7.97 (d, 2 H, Ar-H, *J* = 7.76 Hz), 9.31 (s, 1 H, pyrazole-H5);^13^C NMR (100 MHz, DMSO-d6): δ, ppm: 27.3, 41.9, 55.7, 55.8, 55.9, 79.4, 109.7, 112.4, 115.1, 118.1, 119.2, 122.6, 126.8, 127.5, 129.0, 129.1, 129.3, 130.1, 131.7, 132.6, 137.3, 139.4, 147.3, 148.9, 149.5, 153.3, 155.1, 179.1; MS (EI): m/z = 611 (M^+^). Anal. Calcd. for C_37_H_33_N_5_O_4_ (611.70): C, 72.65; H, 5.44; N, 11.45. Found: C, 72.77; H, 5.58; N, 11.60.

#### 1-(1-(4-Chlorophenyl)-8,9-dimethoxy-1,5,6,10b-tetrahydro-[1,2,4]triazolo[3,4-*a*]isoquinolin-3-yl)-3-(1,3-diphenyl-1*H*-pyrazol-4-yl)prop-2-en-1-one


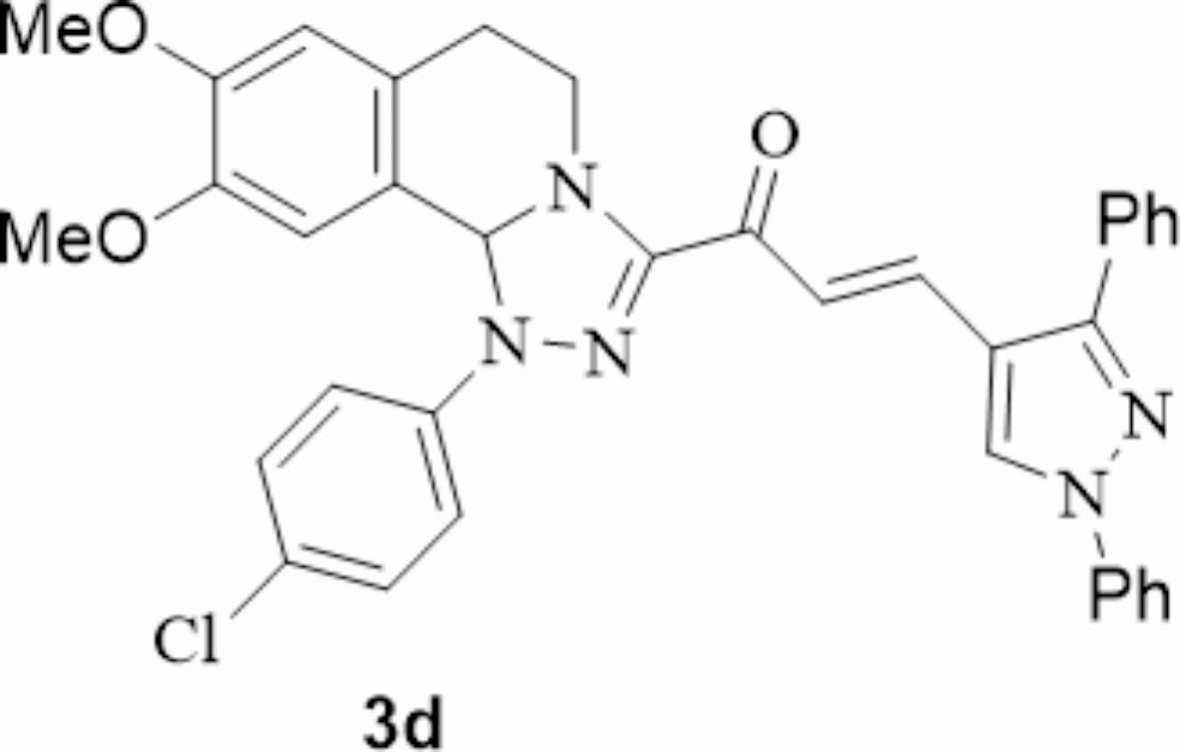


Yield: (84%) as a pale-yellow solid (from ethanol / dioxane); m.p 216–218 °C. IR (KBr, cm^− 1^): 1668 (CO); ^1^H NMR (400 MHz, DMSO-d6): δ, ppm: 2.64–2.83 (m, 2 H, H6), 3.47 (s, 3 H, OMe), 3.63–3.69 (m, 1 H, H5), 3.72 (s, 3 H, OMe), 4.08–4.14 (m, 1 H, H5), 6.60 (s, 1 H, H10b), 6.79 (s, 1 H, H7), 6.88 (s, 1 H, H10), 7.35–7.43 (m, 5 H Ar-H), 7.53–7.67 (m, 9 H, Ar-H and vinyl-H), 7.98 (d, 2 H, Ar-H, *J* = 7.76 Hz), 9.34 (s, 1 H, pyrazole-H5);^13^C NMR (100 MHz, DMSO-d6): δ, ppm: 27.4, 55.8, 55.9, 77.7, 109.0, 112.4, 116.1, 118.0, 119.2, 122.3, 124.7, 127.5, 127.6, 128.8, 129.0, 129.2, 129.3, 129.5, 130.1, 132.5, 132.9, 139.4, 142.9, 147.6, 149.1, 150.3, 153.5, 179.6; MS (EI): m/z = 616 (M^+^). Anal. Calcd. for C_36_H_30_ClN_5_O_3_ (616.12): C, 70.18; H, 4.91; N, 11.37. Found: C, 70.26; H, 5.08; N, 11.43.

#### 1-(1-(4-Bromophenyl)-8,9-dimethoxy-1,5,6,10b-tetrahydro-[1,2,4]triazolo[3,4-*a*]isoquinolin-3-yl)-3-(1,3-diphenyl-1*H*-pyrazol-4-yl)prop-2-en-1-one


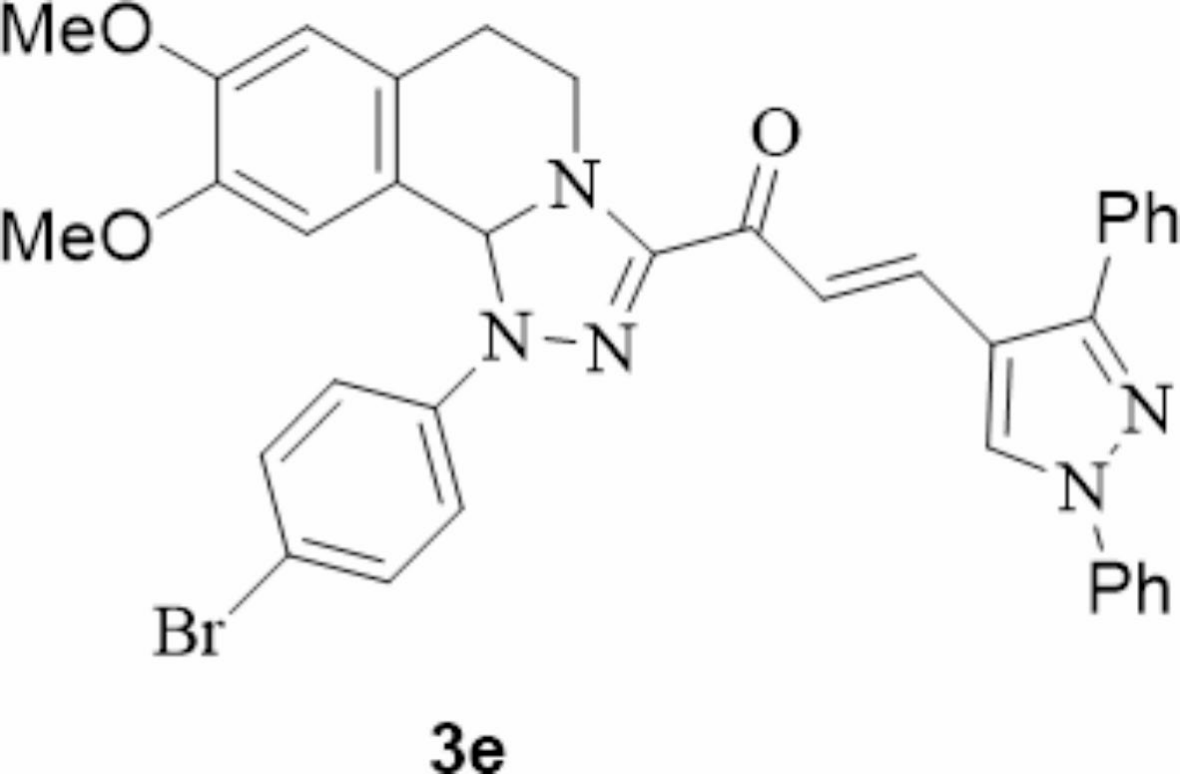


Yield: (83%) as a pale-yellow solid (from ethanol / dioxane); m.p 220–222 °C. IR (KBr, cm^− 1^): 1666 (CO); ^1^H NMR (400 MHz, DMSO-d6): δ, ppm: 2.73–2.89 (m, 2 H, H6), 3.48 (s, 3 H, OMe), 3.92 (br, 4 H, OMe and H5), 4.09–4.31 (m, 1 H, H5), 6.60 (s, 1 H, H10b), 6.80 (s, 1 H, H7), 6.88 (s, 1 H, H10), 7.31–7.99 (m, 14 H, Ar-H and vinyl-H), 7.98 (d, 2 H, Ar-H, *J* = 7.76 Hz), 9.36 (s, 1 H, pyrazole-H5. Anal. Calcd. for C_36_H_30_BrN_5_O_3_ (660.57): C, 65.46; H, 4.58; N, 10.60. Found: C, 65.58; H, 4.67; N, 10.73.

#### 1-(8,9-Dimethoxy-1-(4-nitrophenyl)-1,5,6,10b-tetrahydro-[1,2,4]triazolo[3,4-*a*]isoquinolin-3-yl)-3-(1,3-diphenyl-1*H*-pyrazol-4-yl)prop-2-en-1-one


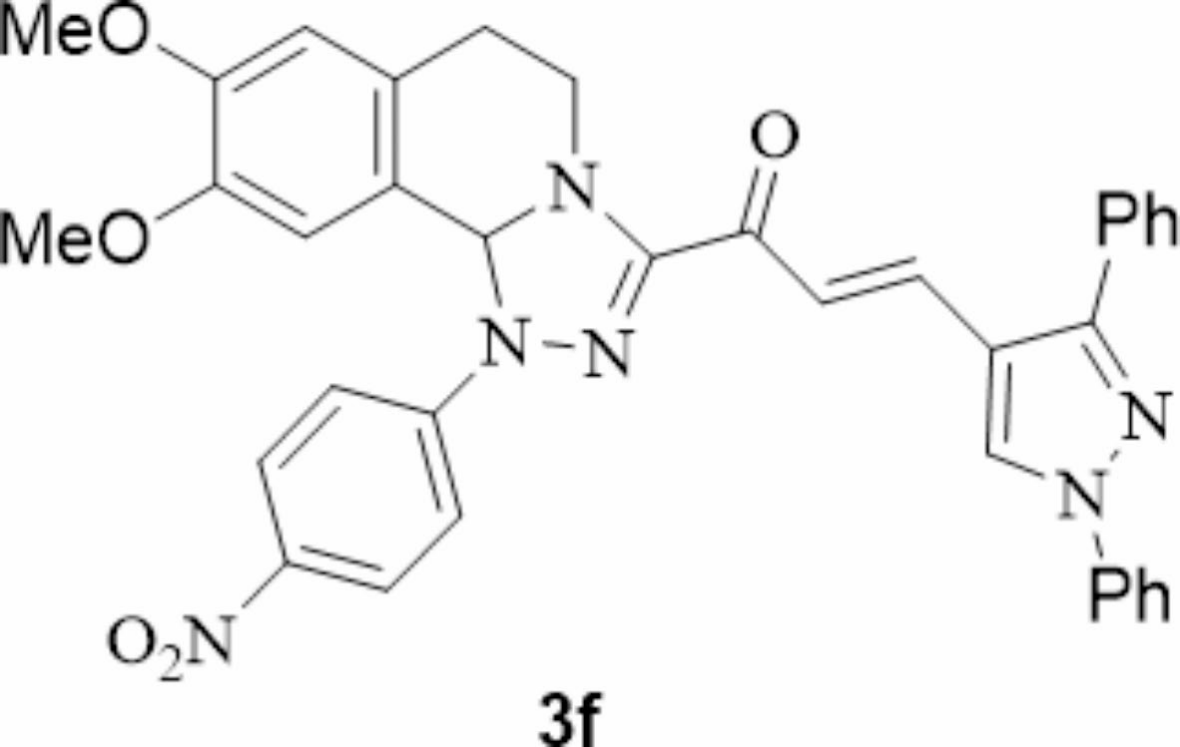


Yield: (87%) as a pale-yellow solid (from ethanol / dioxane); m.p 224–226 °C. IR (KBr, cm^− 1^): 1668 (CO); ^1^H NMR (400 MHz, DMSO-d6): δ, ppm: 2.78–2.89 (m, 2 H, H6), 3.49 (s, 3 H, OMe), 3.74 (s, 3 H, OMe), 3.91–4.07 (m, 2 H, H5), 6.59 (s, 1 H, H10b), 6.86 (s, 1 H, H7), 7.04 (s, 1 H, H10), 7.41–7.72 (m, 12 H Ar-H), 7.98 (d, 2 H, Ar-H, *J* = 8 Hz), 8.27 (d, 2 H, Ar-H, *J* = 8 Hz), 9.40 (s, 1 H, pyrazole-H5);^13^C NMR (100 MHz, DMSO-d6): δ, ppm: 27.4, 42.0, 56.0, 56.1, 76.6, 108.3, 112.6, 112.9, 117.9, 119.3, 122.0, 126.4, 127.1, 127.7, 128.5, 129.1, 129.4, 130.2, 132.4, 134.4, 139.3, 139.6, 147.7, 148.7, 149.3, 152.0, 153.7, 180.1; MS (EI): m/z = 626 (M^+^). Anal. Calcd. for C_36_H_30_N_6_O_5_ (626.67): C, 69.00; H, 4.83; N, 13.41. Found: C, 69.14; H, 4.95; N, 13.56.

### Computational chemistry

Ligands were docked to the X-ray crystal structure of EGFR kinase (PDB: 1M17, resolution 1.71 Å)^64^. Tautomeric and ionization states of EGFR kinase amino acid residues at pH 7.4 were assigned using MOE 2009. Similarly, ligands were modelled in their ionized forms at physiological pH. Docking was performed with the Tringale matcher placement method of MOE using the London dG scoring function with rigid fit as the refinement method. The EGFR kinase inhibitors were docked into the pocket which was occupied by Erlotinib in the X-ray crystal structure of EGFR kinase^75^. Rescoring of poses used the molecular mechanics (MM)/Generalized Born/volume integral (GBVI) potential^76^.

### Biological activity evaluation

#### Cytotoxic activity by MTT assay

Cell line cells were purchased from the American Type Culture Collection (ATCC, Manassas, VA) and were cultured according to the supplier’s instructions. Cells were cultivated in Dulbecco’s Modified Eagle’s Medium (DMEM) (Lonza) supplemented with 10% fetal bovine serum (FBS), 1% penicillin-streptomycin mixture, and 1% L-glutamine at 37 °C under 5% CO_2_. The media was changed every 48 h, and the cells were split when 80–90% confluence was reached. Cell viability was evaluated by MTT assay. The reduction of yellow tetrazolium salt (3-(4,5-dimethylthiazol-2-yl)-2,5-diphenyltetrazolium bromide (MTT) to purple formazan crystals is done by mitochondria. In brief, a cell concentration of 5 × 10^3^ cells/well was cultured in 96-well microtiter plates at 37°C for 24 h. Cells were treated with different concentrations of the compounds (100, 50, 25, 12.5, 6.25, 3.125, 1.56, 0.781 µM). Untreated cells were used as the negative control, while 5-Fluorouracil and three EGFR inhibitors drugs: Lapatinib, Erlotinib and Gefitinib were used as a positive control for comparison. Lapatinib, Gefitinib, and Erlotinib were obtained from Targetmol, USA. The cells were washed twice with phosphate-buffered saline after 24 h of incubation. Finally, 2.5 µM MTT was added, and the 96-well plates were incubated for 4 h more before stopping the reaction with 10% sodium dodecyl sulphate (SDS). The absorbance was measured at 595 nm and the following formula was used to calculate cell viability percentage^60^.

$${\text{Cell Viability}} {\%}=\frac{{\text{OD of treated cells}}-{\text{OD of blank}}}{{\text{OD of control cells}}-{\text{OD of blank}}} \times 100$$

#### Apoptosis and cell cycle analysis by Annexin V-FITC assay60

Quantitation of DNA content was evaluated using the Annexin V-FITC Apoptosis Detection kit (BioVision, CA, USA). Dual staining for Annexin-V and PI allows the discrimination between live cells, necrotic cells, and cells in different phases of apoptosis. A549 cancer cells were treated with **3e** and **3f** IC_50_ values (1.45 and 0.72 µM, respectively) for 24 h. Then, cells were collected and washed with 1X phosphate-buffered saline. The cells were stained in the dark with annexin V-FITC and propidium iodide in a binding buffer and analyzed using the flow cytometer. The cell cycle profiles were analysed using MultiCycle software (Phoenix Flow Systems, San Diego, CA)^60^.

#### Real-time PCR analysis

The real-time polymerase chain reaction (RT-qPCR) technique was used to evaluate the gene expression. A panel of eight genes (P53, BAX, Caspase-3, Caspase-8, Caspase-9, MMP1, CDK4, and Bcl2) was chosen to estimate the compounds’ ability to induce apoptosis. A549 cancer cells were treated with **3e** and **3f** compounds for 24 h before total RNA extraction. While untreated A549 cancer cells were utilized as a negative control. One-step RT-qPCR was performed using the iScript™ One-Step RT-PCR kit with SYBR^®^. The primer pairs sequences for each gene used are mentioned in Table 7. Control reactions were formed with an RNA template or without the reverse transcriptase enzyme.

**Table 7:** Sequences of primers used for each gene in the RT-qPCR analysis.

Gene	Primer	
P53	F	5′-GCCCAACAACACCAGCTCCT-3′
	R	5′-CCTGGGCATCCTTGAGTTCC-3′
Bax	F	5′-TTCCGAGTGGCAGCTGAGATGTTT-3′
	R	5′-TGCTGGCAAAGTAGAAGAGGGCAA-3′
Caspase 3	F	5′-TTCATTATTCAGGCCTGCCGAGG-3′
	R	5′-TTCTGACAGGCCATGTCATCCTCA-3′
Caspase 8	F	5′-ACAATGCCCAGATTTCTCCCTAC-3′
	R	5′-CAGACAGTATCCCCGAGTTTG-3′
Caspase 9	F	5′-TCAGTGACGTCTGTGTTCAGGAGA-3′
	R	5′-TTGTTGATGATGAGGCAGTAGCCG-3′
MMP1	F	5′- CTGGCCACAACTGCCAAATG-3′
	R	5′-CTGTCCCTGAACAGCCCAGTACTTA-3′
CDK4	F	5′- TCGAAAGCCTCTCTTCTGTG-3′
	R	5′-TACATCTCGAGGCCAGTCAT- 3′
Bcl2	F	5′-CATGCCAAGAGGGAAACACCAGAA-3′
	R	5′-GTGCTTTGCATTCTTGGATGAGGG-3′
β-actin gene	F	5′-TTCCTGGGCATGGAGTC-3′
	R	5′-CAGGTCTTTGCGGATGTC-3′

#### EGFR inhibition assay

EGFR inhibition assay was performed on A549 cancer cells using cloud clone product SEA757Hu 96 Ki, according to manufacturer’s instructions protocol. The homogeneous time-resolved fluorescence assay method was used to assess the inhibitory activity of **3e** and **3f** against EGFR^WT^, EGFR^T790M^ and EGFR^L858R^. The EGFR proteins (EGFR^WT^, EGFR^T790M^ and EGFR^L858R^) were obtained from Signalchem (BC V6V 2J2, Canada). ATP and other chemicals were obtained from Sigma. The compounds, along with Lapatinib as a reference drug, were incubated with each EGFR protein and substrate for 5 min, after which ATP was added. After 30 min, the reaction was stopped by adding detection reagents containing EDTA. The readings were taken after 1 h, and the IC_50_, the compound concentration required to kill 50% of the cell population, was determined by GraphPad Prism 4.0 (San Diego, CA, USA).

### Data treatment and statistical analysis

All the experiments were conducted in triplicate and repeated at least three times. The IC_50_ was determined from the Log(inhibitor) vs. normalized response, Variable slope, least squares fit model of GraphPad Prism 4.0, where IC_50_ was reported as means ± SD; statistical significance was evaluated using Student’s t-test and the values were considered significant at *P* < 0.05. The selectivity index of the compounds is calculated as SI = IC_50_ of the pure compound in a normal cell line/IC_50_ of the same pure compound in the cancer cell line.

## Electronic supplementary material

Below is the link to the electronic supplementary material.

Supplementary Material 1

## Data Availability

The data that support the findings of this study are available from the corresponding author upon reasonable request.
