# Supplementary material for: Design, in silico studies and biological evaluation of novel chalcones tethered triazolo[3,4-a]isoquinoline as EGFR inhibitors targeting resistance in non-small cell lung cancer
Source: Sci Rep. 2024 Nov 4;14:26647. doi: 10.1038/s41598-024-76459-x (PMC11535068; doi:10.1038/s41598-024-76459-x)
Supplement: Supplementary file 1 — Supplementary Material 1 [file 41598_2024_76459_MOESM1_ESM.docx]

**Design, *in silico* studies and biological evaluation of novel chalcones tethered** **triazolo[3,4-*a*]isoquinoline as EGFR inhibitors targeting resistance in non-small cell lung cancer**

Nesma Abdelaal^1^, Mohamed A. Ragheb^2^, Hamdi M. Hassaneen^3^, Emad M. Elzayat^1,4*^, Ismail A. Abdelhamid^3*^

**Affiliation**

^1^Biotechnology Department, Faculty of Science, Cairo University, Egypt.

^2^Department of Chemistry (Biochemistry Division), Faculty of Science, Cairo University, Giza 12613, Egypt.

^3^Chemistry Department, Faculty of Science, Cairo University, Egypt.

^4^Zoology Department, Faculty of Science, Cairo University, Egypt.


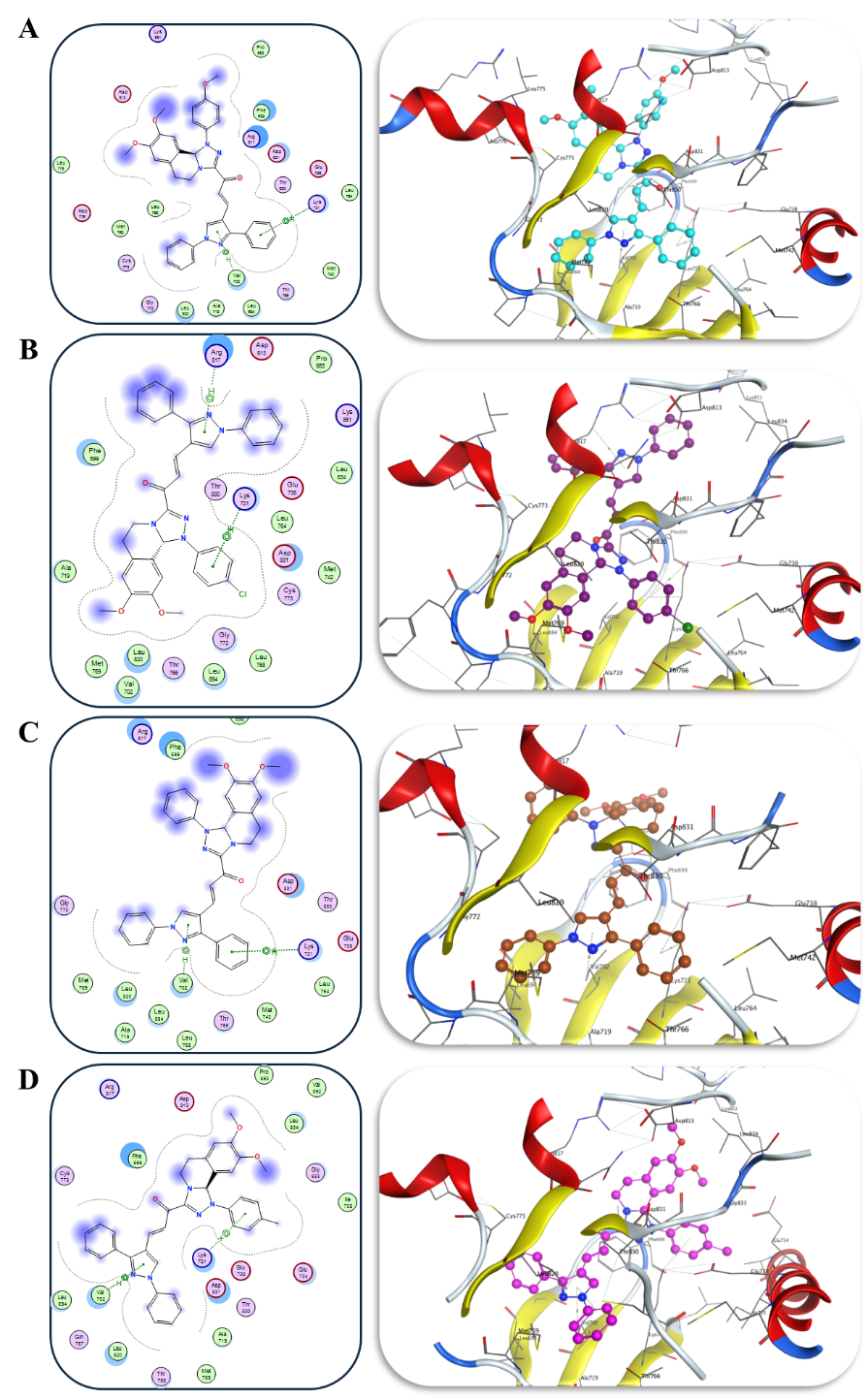


**Figure S1**. Chalcones (**3c, A**; **3d**, **B**; **3a**, **C**; **3b, D**) interactions (2D, left panel; 3D right panel) with EGFR tyrosine kinase ATP binding site (PDB ID: 1M17).


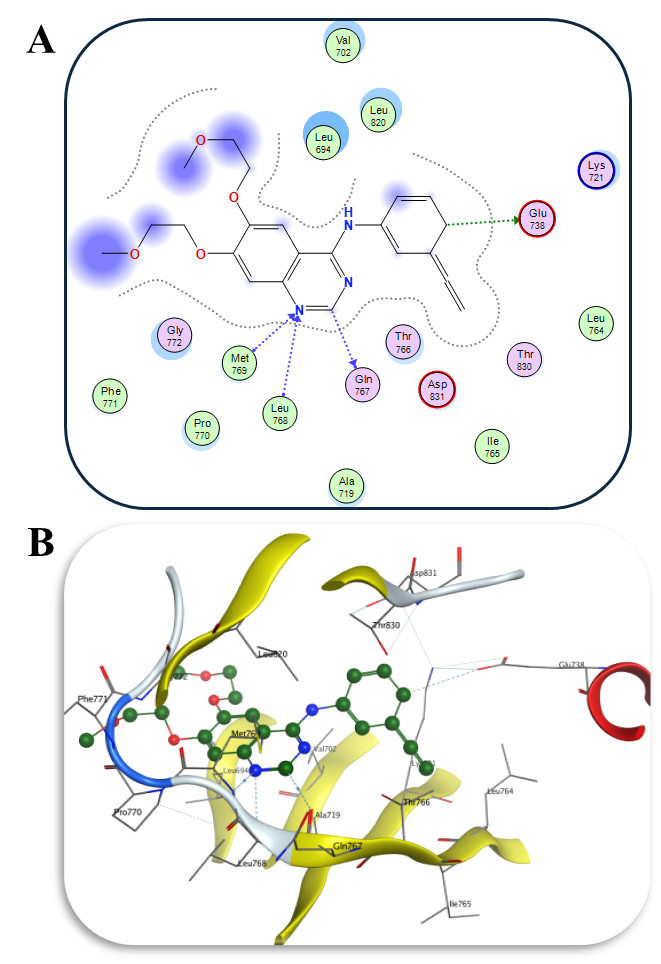


**Figure S2**. Erlotinib, co-crystalized ligand, interaction (2D, upper panel; 3D, lower panel) within EGFR tyrosine kinase ATP binding site (PDB ID: 1M17).

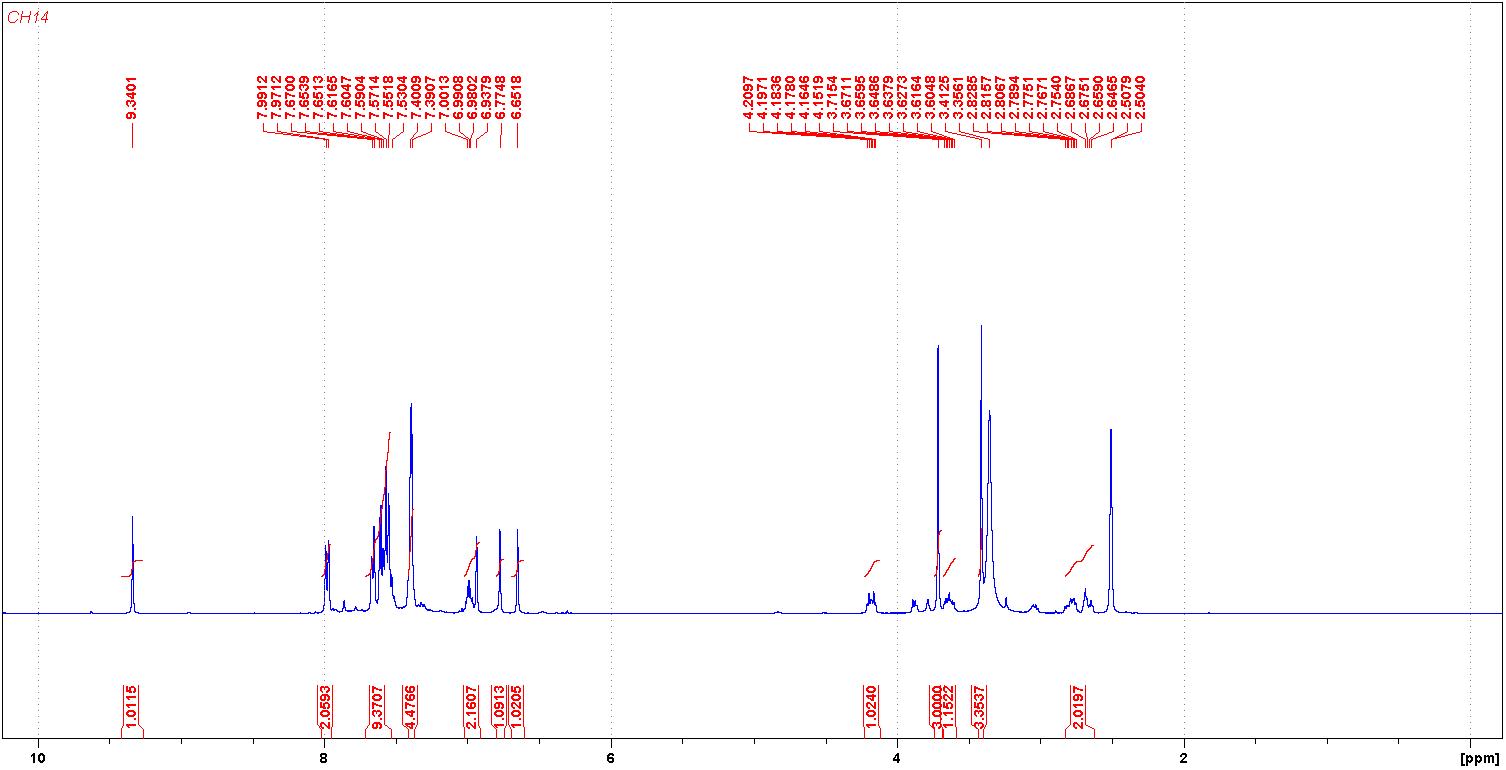


**^1^H NMR of compound 3a**

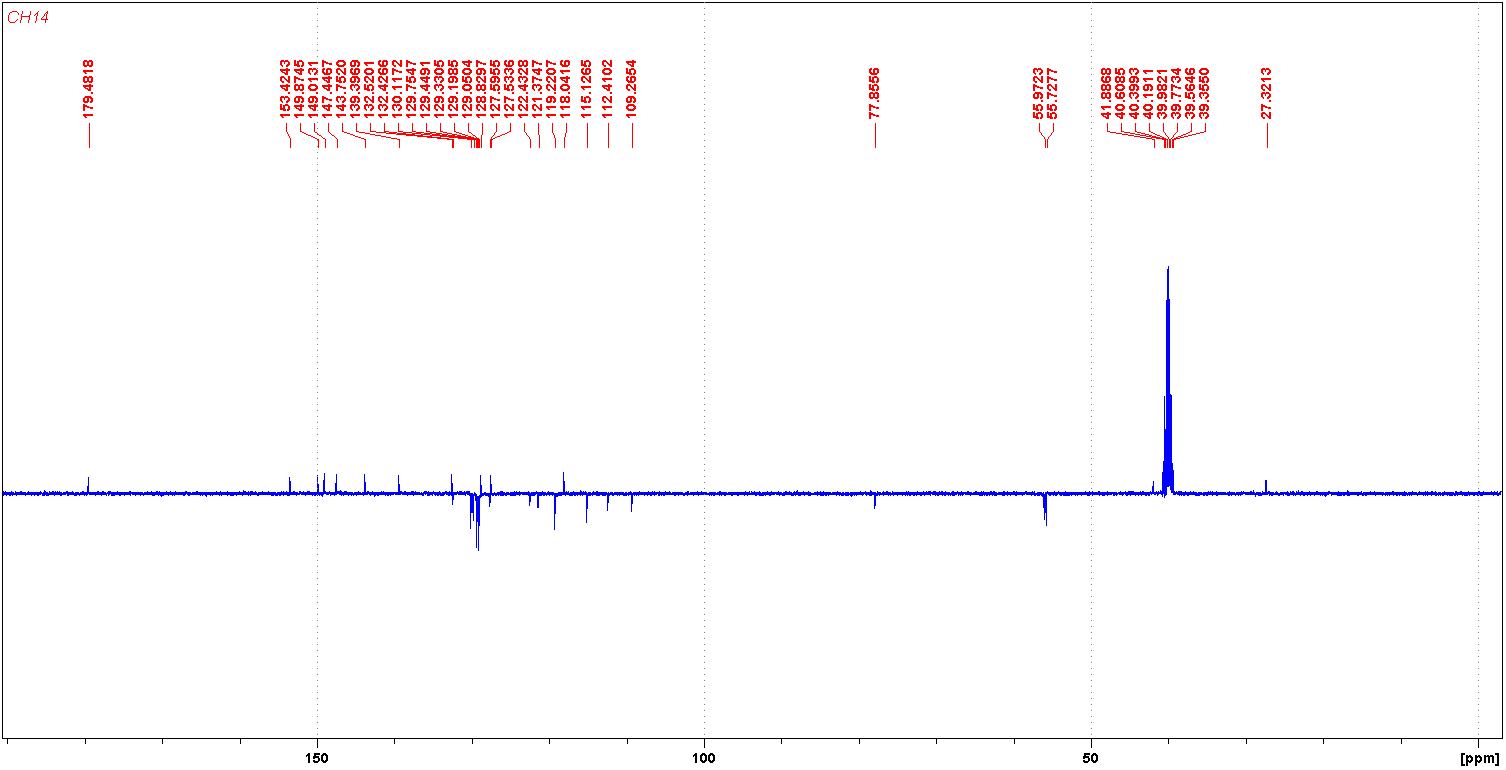


**^13^C NMR of compound 3a**

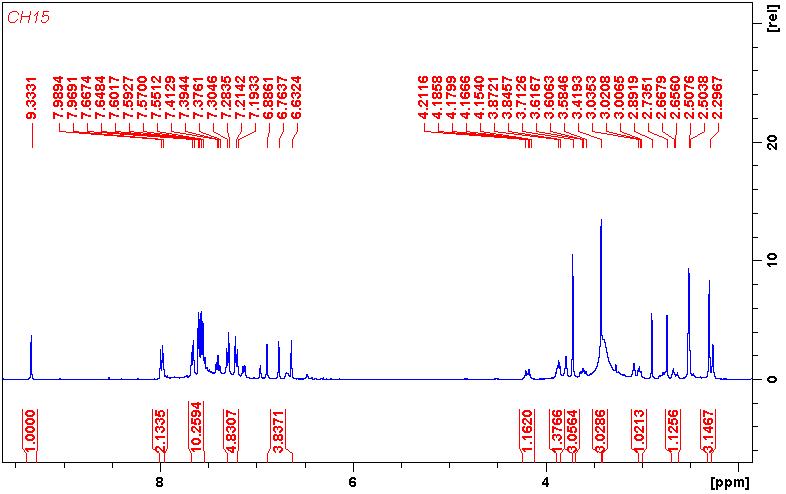


**^1^H NMR of compound 3b**

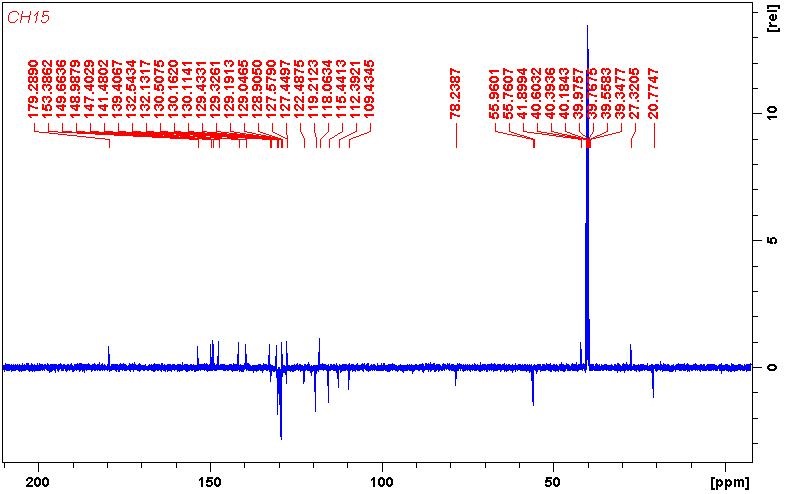


**^13^C NMR of compound 3b**

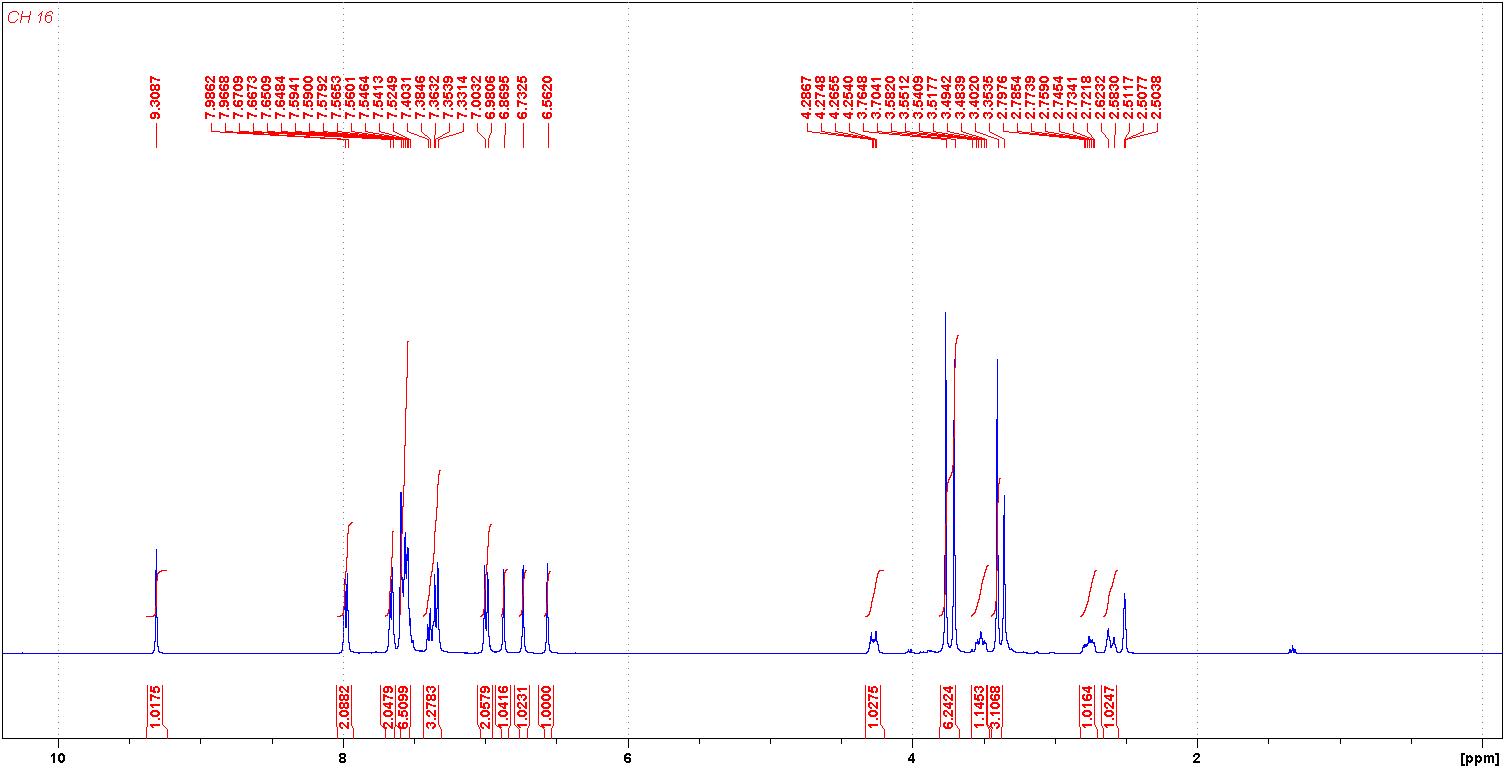


**^1^H NMR of compound 3c**

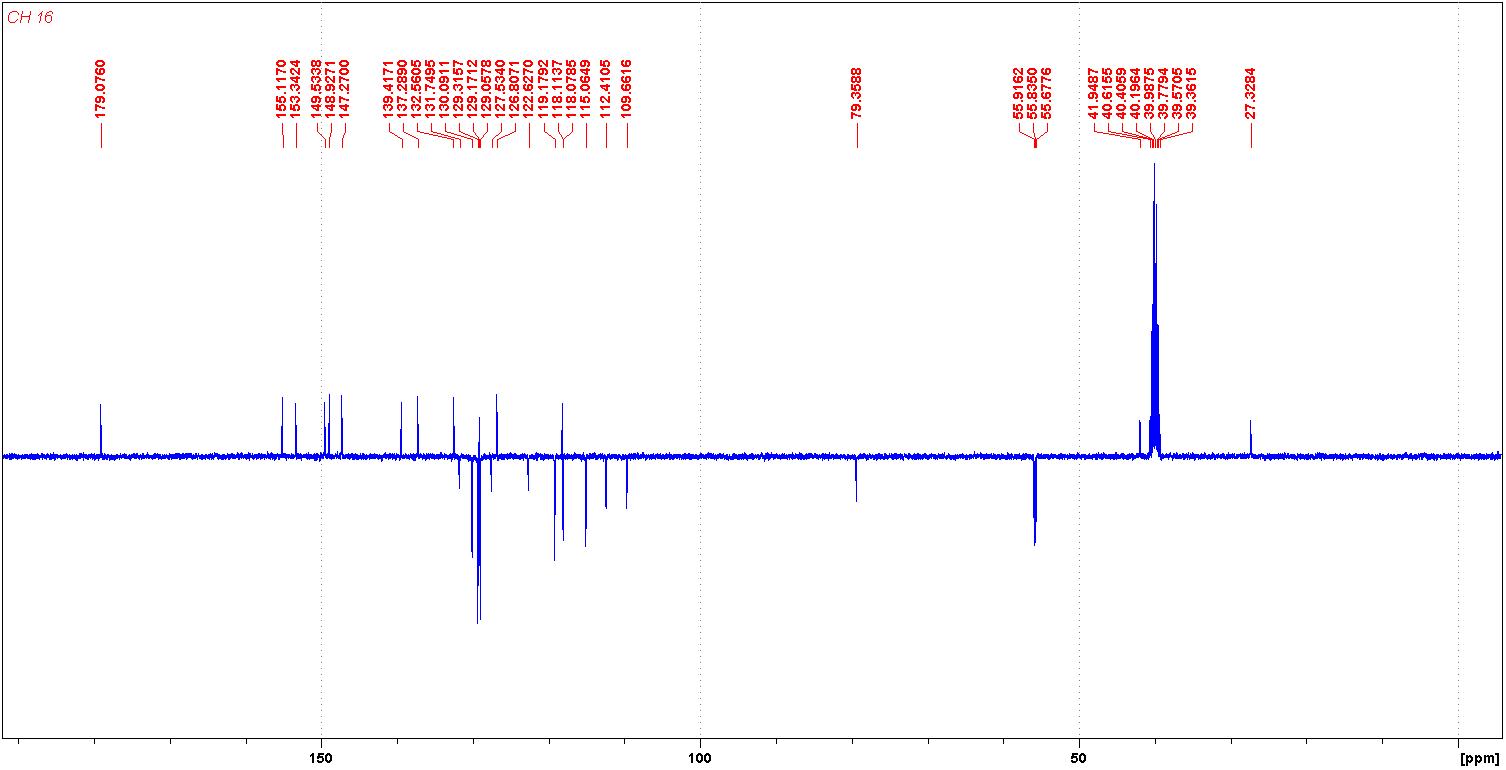


**^13^C NMR of compound 3c**

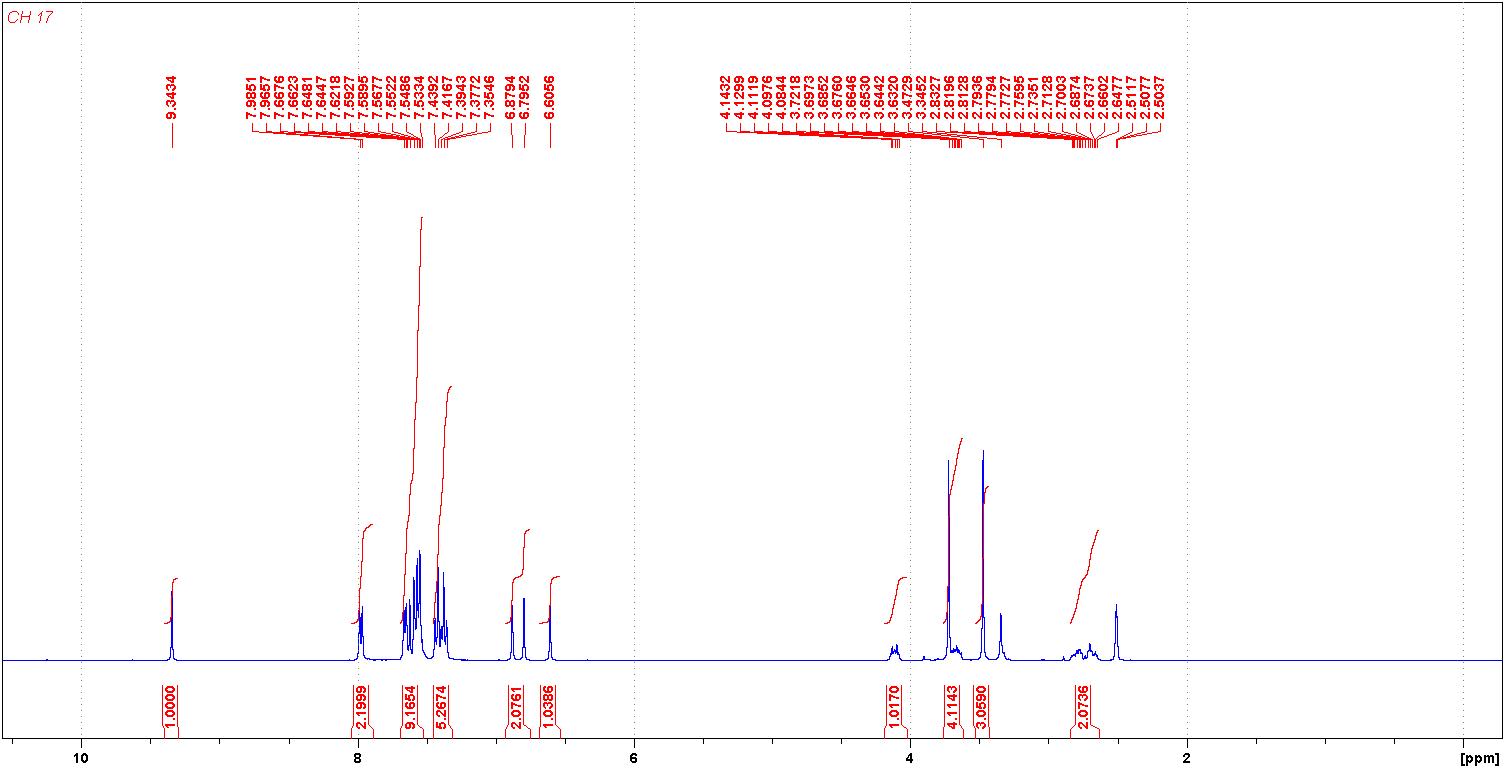


**^1^H NMR of compound 3d**

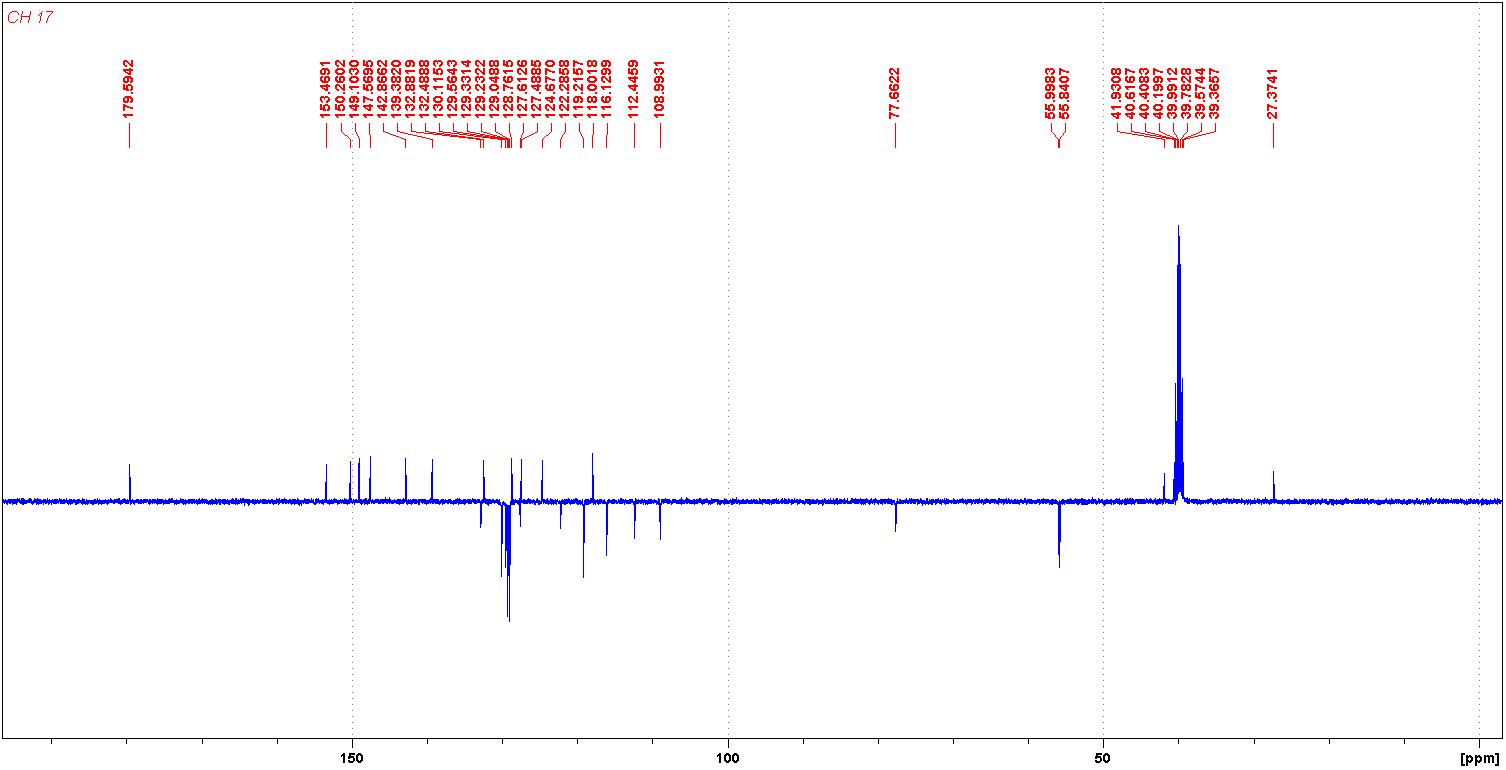


**^13^C NMR of compound 3d**

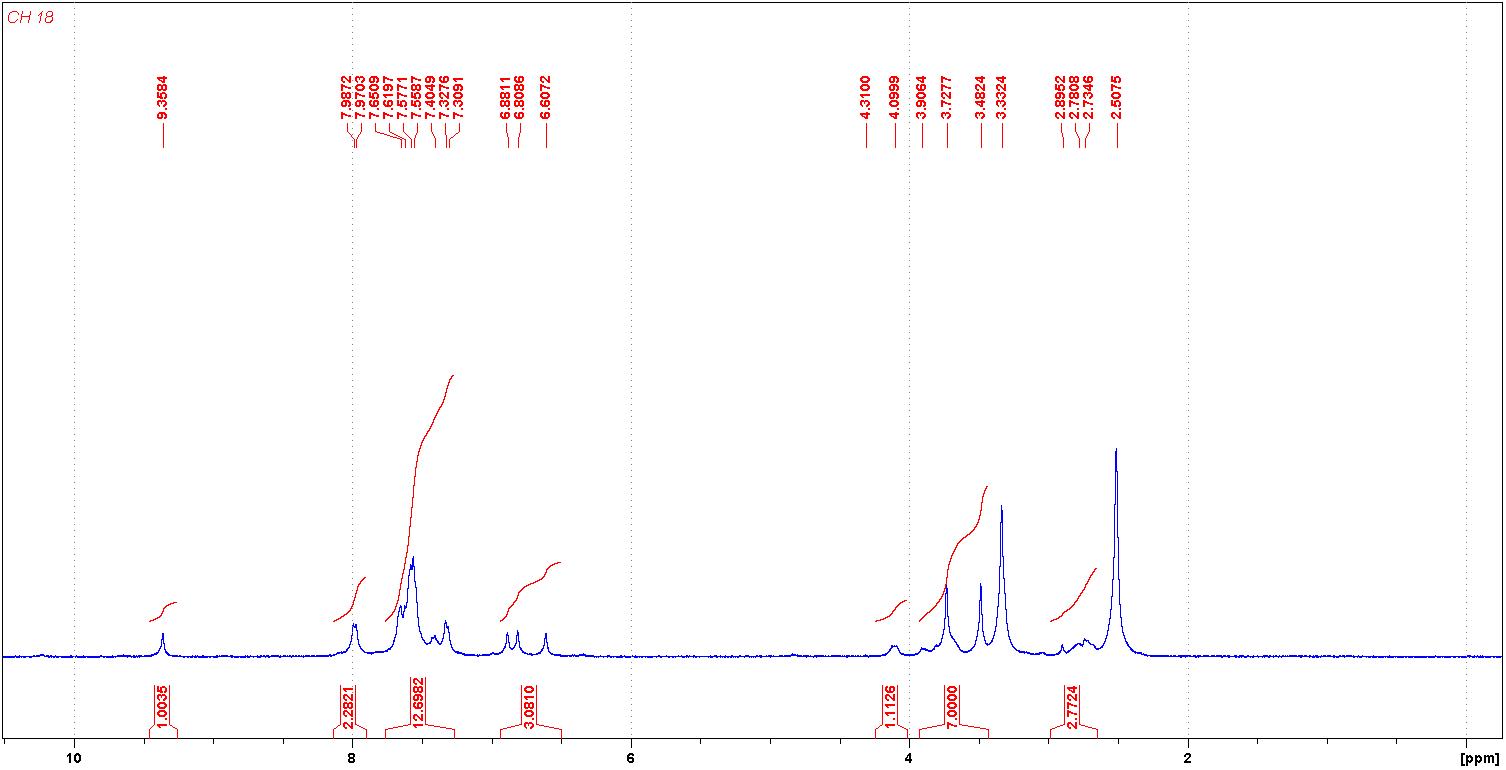


**^1^H NMR of compound 3e**

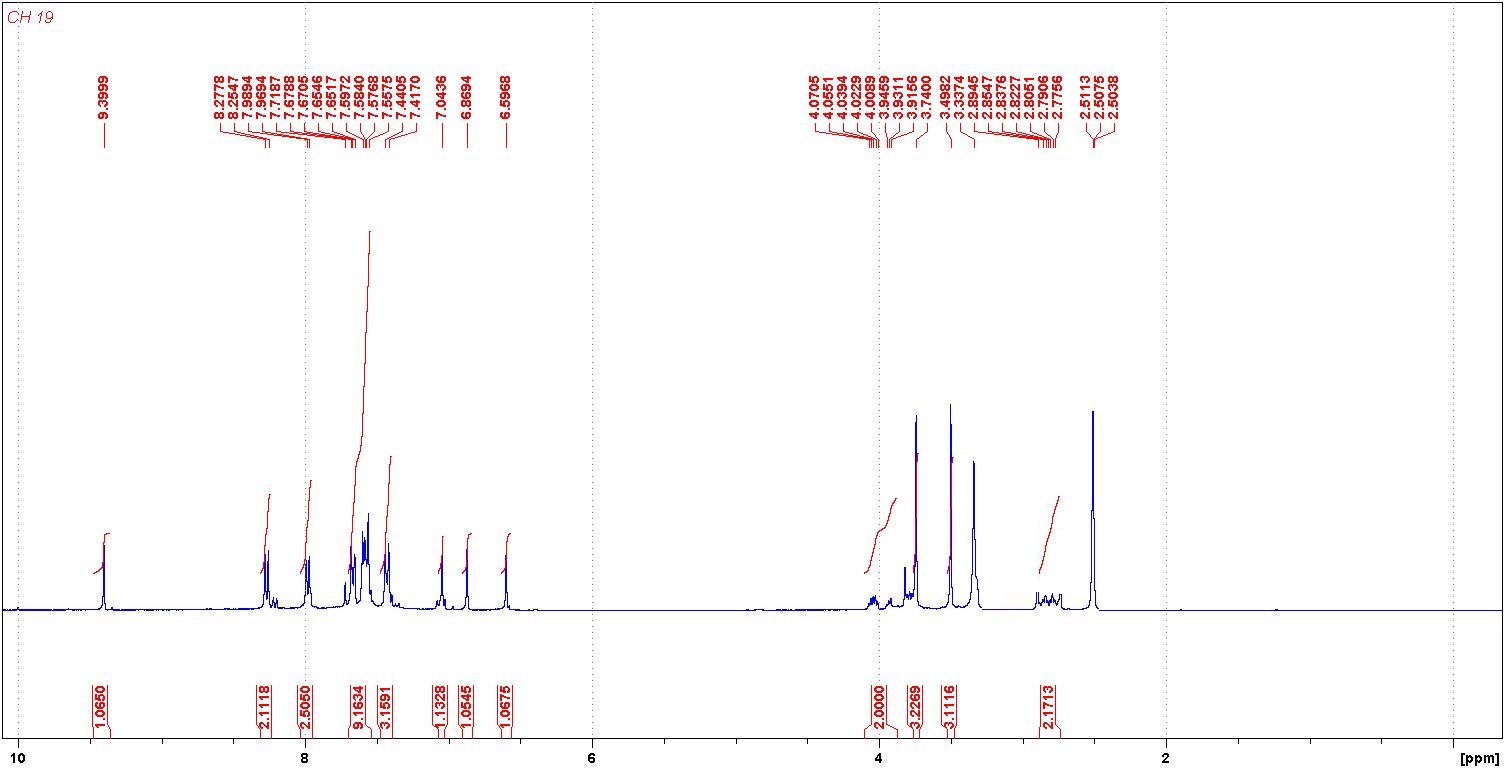


**^1^H NMR of compound 3f**

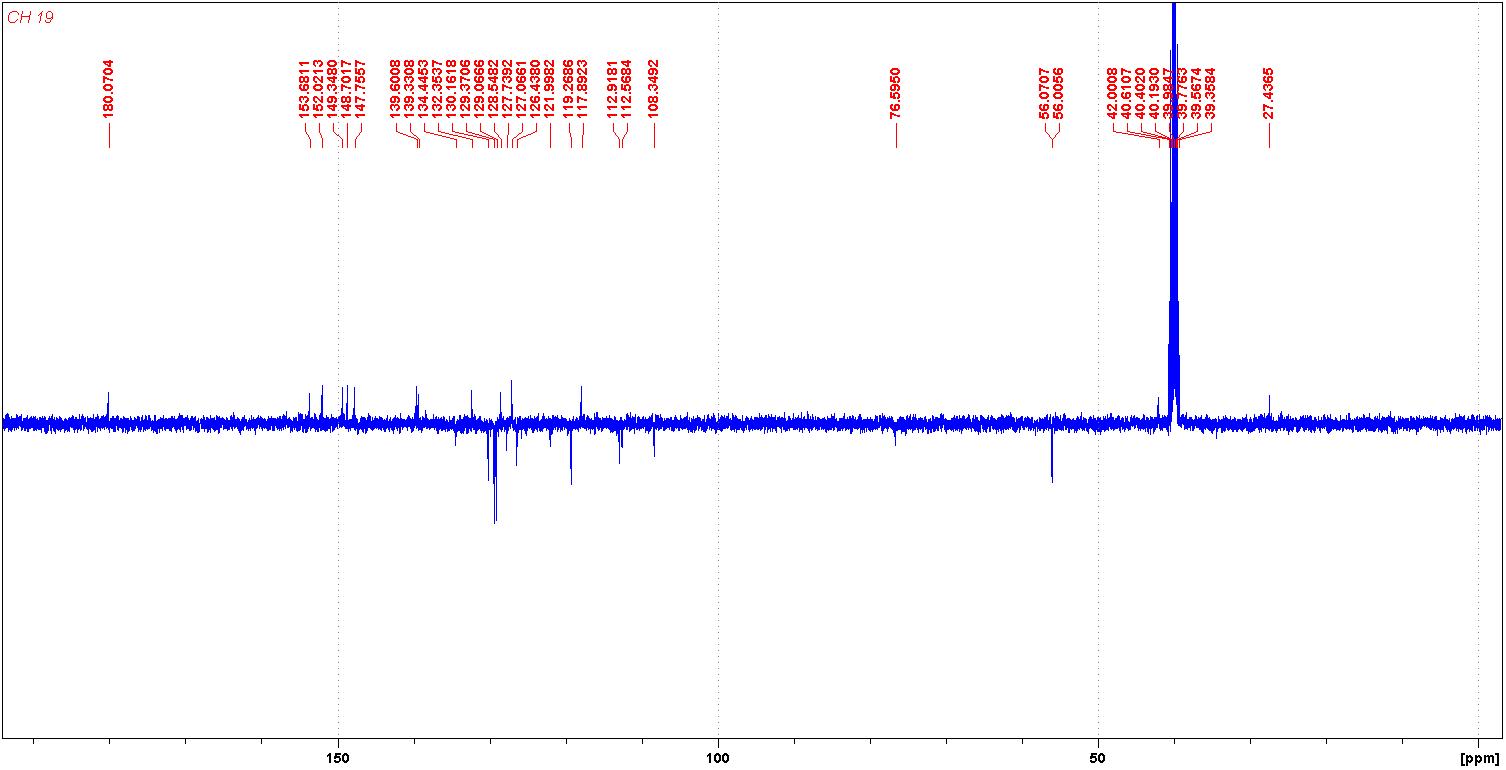


**^13^C NMR of compound 3f**


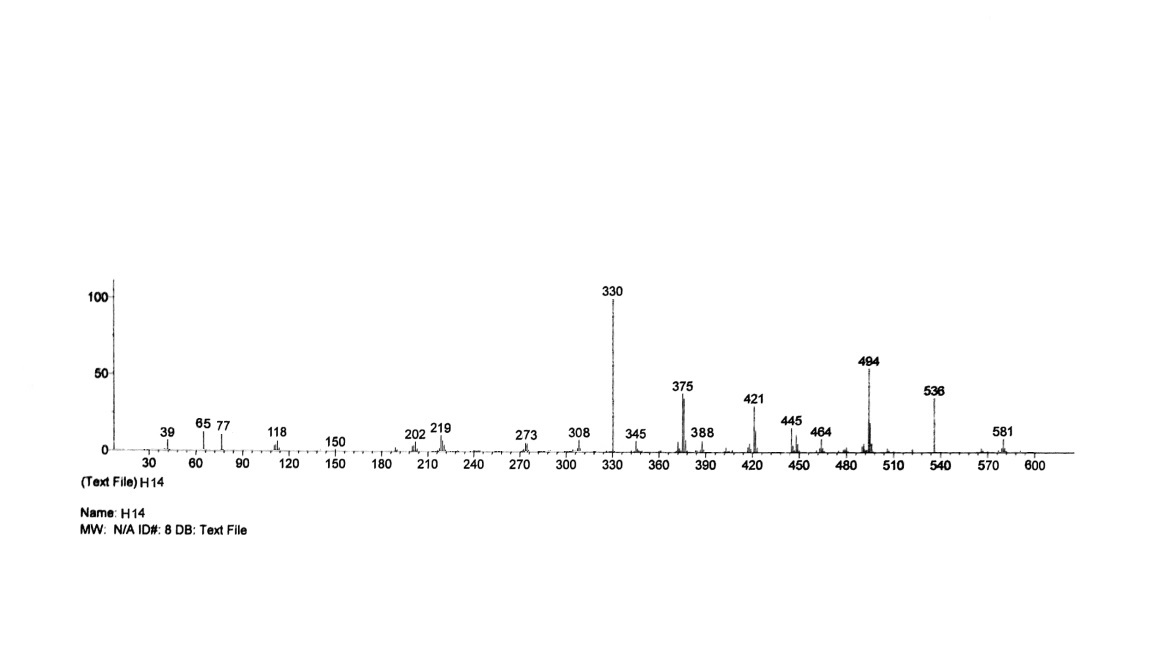


**Mass spectra of compounds 3a**


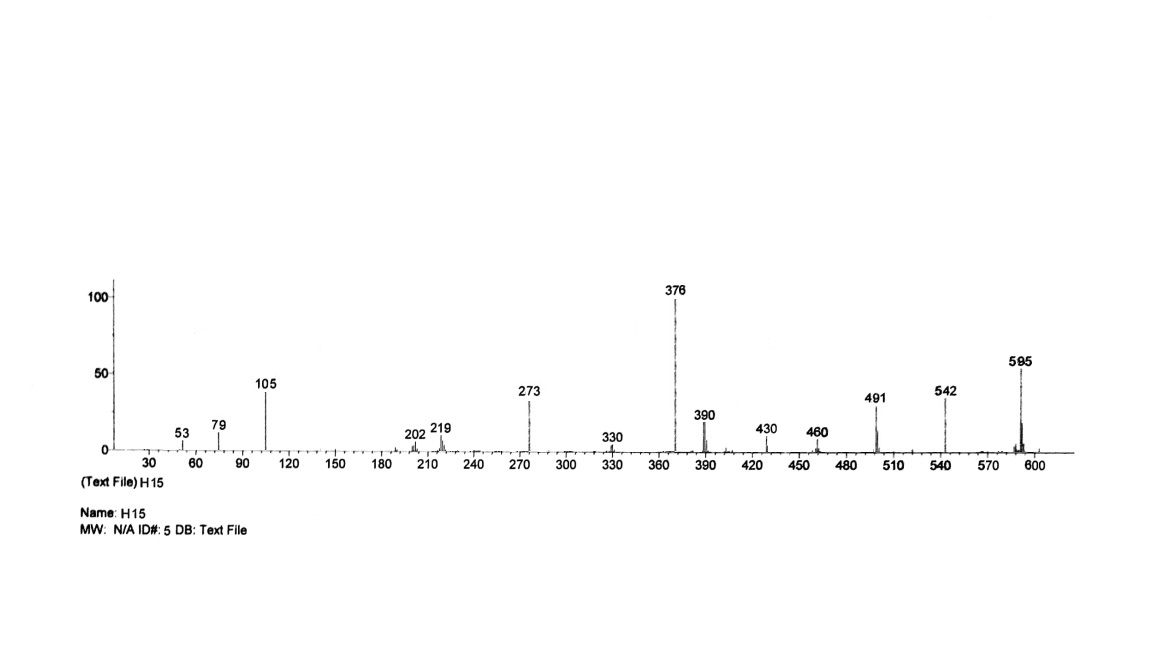


**Mass spectra of compounds 3b**


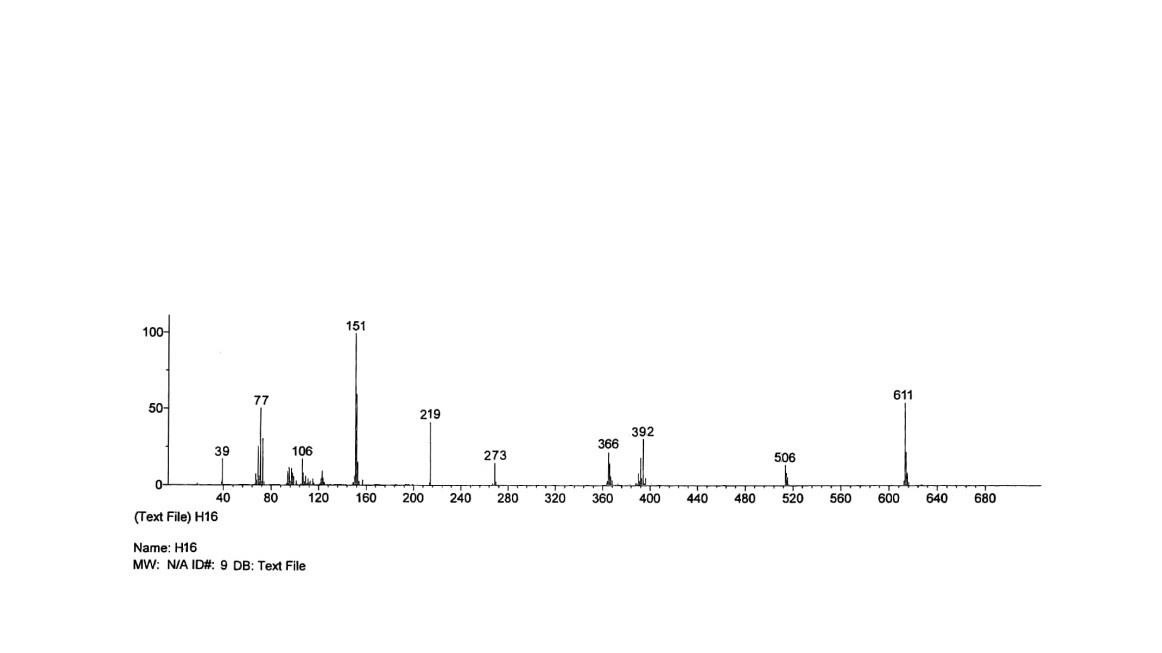


**Mass spectra of compounds 3c**


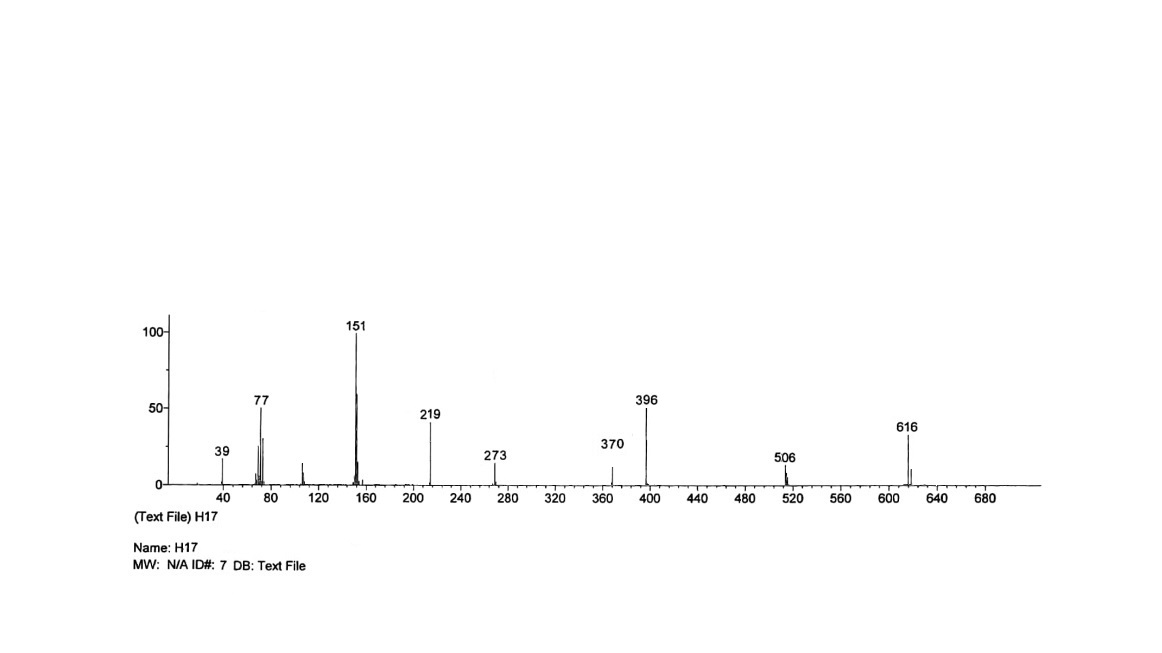


**Mass spectra of compounds 3d**


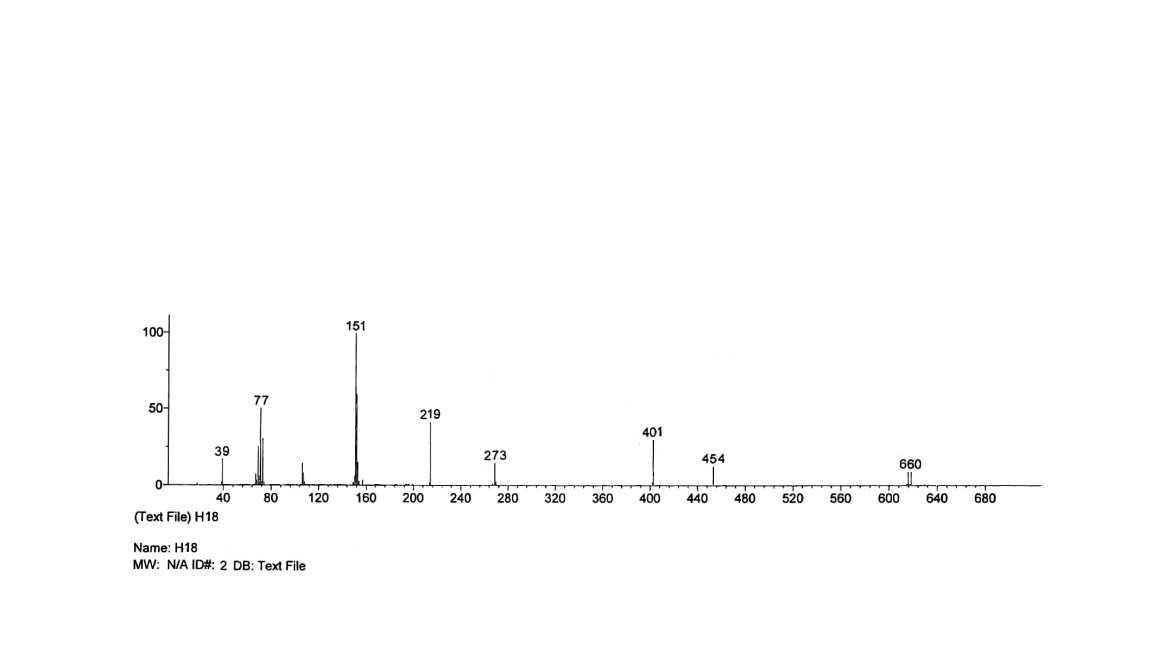


**Mass spectra of compounds 3e**

**
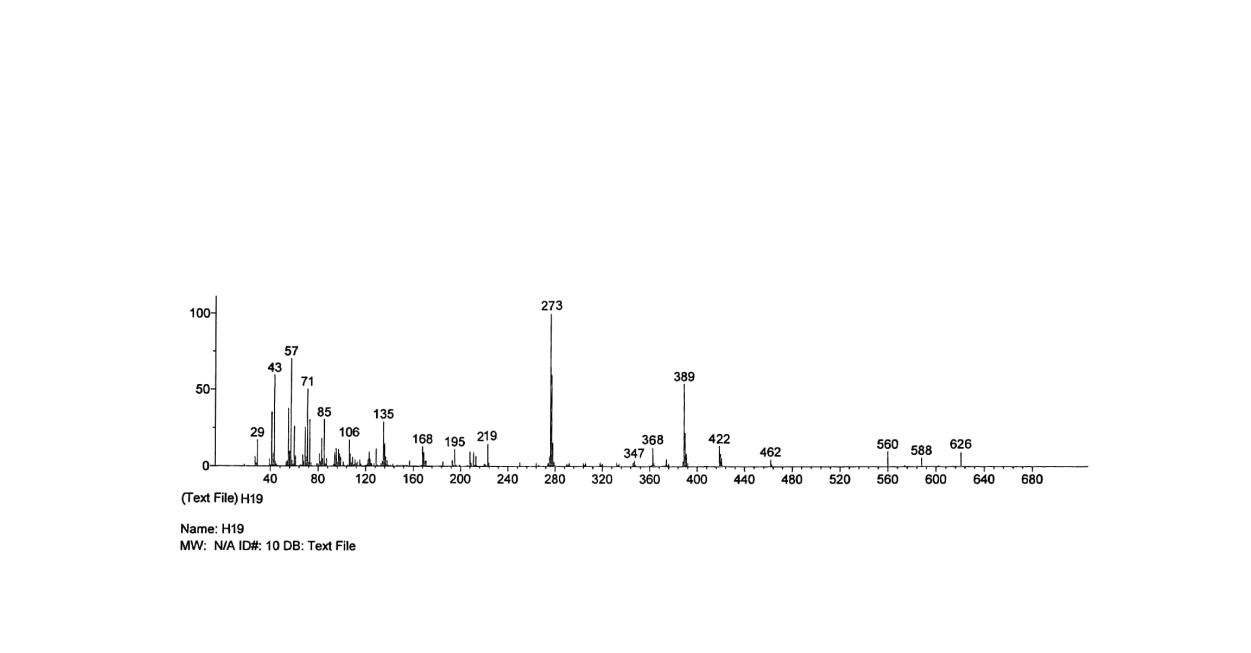
**

**Mass spectra of compounds 3f**
